# Identification and Functional Analysis of *Trypanosoma cruzi* Genes That Encode Proteins of the Glycosylphosphatidylinositol Biosynthetic Pathway

**DOI:** 10.1371/journal.pntd.0002369

**Published:** 2013-08-08

**Authors:** Mariana S. Cardoso, Caroline Junqueira, Ricardo C. Trigueiro, Hosam Shams-Eldin, Cristiana S. Macedo, Patrícia R. Araújo, Dawidson A. Gomes, Patrícia M. Martinelli, Jürgen Kimmel, Philipp Stahl, Sebastian Niehus, Ralph T. Schwarz, José O. Previato, Lucia Mendonça-Previato, Ricardo T. Gazzinelli, Santuza M. R. Teixeira

**Affiliations:** 1 Departamento de Bioquímica e Imunologia, Universidade Federal de Minas Gerais, Belo Horizonte, Minas Gerais, Brazil; 2 Centro de Pesquisas René Rachou, Fundação Oswaldo Cruz, Belo Horizonte, Minas Gerais, Brazil; 3 Institut für Virologie – AG Parasitologie, Philipps-Universität Marburg, Marburg, Germany; 4 Instituto de Biofísica Carlos Chagas, Universidade Federal do Rio de Janeiro, Rio de Janeiro, Rio de Janeiro, Brazil; 5 Departamento de Morfologia, Universidade Federal de Minas Gerais, Belo Horizonte, Minas Gerais, Brazil; Durham University, United Kingdom

## Abstract

**Background:**

*Trypanosoma cruzi* is a protist parasite that causes Chagas disease. Several proteins that are essential for parasite virulence and involved in host immune responses are anchored to the membrane through glycosylphosphatidylinositol (GPI) molecules. In addition, *T. cruzi* GPI anchors have immunostimulatory activities, including the ability to stimulate the synthesis of cytokines by innate immune cells. Therefore, *T. cruzi* genes related to GPI anchor biosynthesis constitute potential new targets for the development of better therapies against Chagas disease.

**Methodology/Principal Findings:**

*In silico* analysis of the *T. cruzi* genome resulted in the identification of 18 genes encoding proteins of the GPI biosynthetic pathway as well as the inositolphosphorylceramide (IPC) synthase gene. Expression of GFP fusions of some of these proteins in *T. cruzi* epimastigotes showed that they localize in the endoplasmic reticulum (ER). Expression analyses of two genes indicated that they are constitutively expressed in all stages of the parasite life cycle. *T. cruzi* genes *TcDPM1*, *TcGPI10* and *TcGPI12* complement conditional yeast mutants in GPI biosynthesis. Attempts to generate *T. cruzi* knockouts for three genes were unsuccessful, suggesting that GPI may be an essential component of the parasite. Regarding *TcGPI8*, which encodes the catalytic subunit of the transamidase complex, although we were able to generate single allele knockout mutants, attempts to disrupt both alleles failed, resulting instead in parasites that have undergone genomic recombination and maintained at least one active copy of the gene.

**Conclusions/Significance:**

Analyses of *T. cruzi* sequences encoding components of the GPI biosynthetic pathway indicated that they are essential genes involved in key aspects of host-parasite interactions. Complementation assays of yeast mutants with these *T. cruzi* genes resulted in yeast cell lines that can now be employed in high throughput screenings of drugs against this parasite.

## Author Summary

Chagas disease, considered one of the most neglected tropical diseases, is caused by the blood-borne parasite *Trypanosoma cruzi* and currently affects about 8 million people in Latin America. *T. cruzi* can be transmitted by insect vectors, blood transfusion, organ transplantation and mother-to-baby as well as through ingestion of contaminated food. Although *T. cruzi* causes life-long infections that can result in serious damage to the heart, the two drugs currently available to treat Chagas disease, benznidazole and nifurtimox, which have been used for more than 40 years, have proven efficacy only during the acute phase of the disease. Thus, there is an urgent need to develop new drugs that are more targeted, less toxic, and more effective against this parasite. Here we described the characterization of *T. cruzi* genes involved in the biosynthesis of GPI anchors, a molecule responsible for holding different types of glycoproteins on the parasite membrane. Since GPI anchored proteins are essential molecules *T. cruzi* uses during infection, besides helping understand how this parasite interacts with its host, this work may contribute to the development of better therapies against Chagas disease.

## Introduction

Glycosylphosphatidylinositol (GPI) is an abundant component of the plasma membrane of protist parasites. In most eukaryotic cells, GPIs are found as free molecules or as lipid anchor for proteins that are bound to the cell surface [1]. They are complex molecules that are synthesized in the ER by sequential addition of sugar residues and other substituents, e.g. ethanolamine-phosphate, to the phosphatidylinositol (PI) precursor and transported to the cell surface, as a free GPI also known as GIPL (glycoinositolphospholipid) or linked to the C-terminus of a protein that contains a GPI signal sequence [2]. Numerous studies with different parasites clearly show that GIPLs and GPI-anchored proteins play important roles in different processes related to host-parasite interaction. Also, it has been suggested that, because of the existence of differences in the structure of GPI from several parasite species as well as between GPIs of the parasite and their host cells [2], [3], [4], these molecules constitute promising targets for studies towards the development of new anti-microbial drugs [5].

*Trypanosoma cruzi* is a parasitic protist that causes Chagas disease, an illness not only prevalent in Latin America, where an estimated 8 million people are infected, but a worldwide health issue for which there is an urgent need for the development of new chemotherapeutic agents and more effective prophylactic methods (www.who.int/topics/chagas_disease/en/). The surface of *T. cruzi* is covered by a large amount of GPI-anchored proteins whose structure and chemical composition have been extensively studied [6] and are expressed in all developmental stages of the parasite life cycle [3], [7]. Analysis of the *T. cruzi* genome indicated that 12% of the parasite genes encode proteins anchored by GPI, a percentage that is much higher when compared with other organisms [8]. Many of these proteins play important roles in the invasion process and, since they show varying sequences, they could also participate in the processes responsible for evasion of the host immune response [9], [10]. Two main components of the *T. cruzi* surface, the *trans*-sialidases and mucins, which act, respectively, as enzymes responsible for the transfer and acceptors for sialic acid molecules, are GPI-anchored glycoproteins [11]. It has also been demonstrated that *T. cruzi* GPI-anchored mucins as well as free GPI anchors act as potent pro-inflammatory agents that are recognized by Toll like receptors [12] and, because of their role in activating the innate immune response, they have been used as adjuvants in immunization protocols [13].

In *Saccharomyces cerevisiae*, biosynthesis of GPI is essential for cell growth and occurs in eleven steps beginning with the transfer of a molecule of N-acetyl-glucosamine (GlcNAc) from UDP-GlcNAc to PI [14], [15]. After the addition of mannose molecules using dolichol-P-mannose as a donor, followed by the transfer of ethanolamine-phosphate (EtNP) to the third mannose residue, GPI is transferred to proteins that have a predicted GPI addition signal at their C-terminal end, in a reaction catalyzed by the GPI-transamidase complex [16]. Genes encoding enzymes involved in GPI pathway from various organisms, including protist parasites such as *Trypanosoma brucei*, *Leishmania mexicana* and *Plasmodium falciparum* have been cloned and their products characterized by functional complementation in mammalian cells and in yeast mutants [17], [18], [19], [20]. Although the main structure of GPI is conserved in all organisms, several studies have shown differences in the biosynthetic pathway and additional modifications to GPI structures present in mammalian and parasite cells [2], [3], [4]. Substrate analogues of enzymes of the GPI biosynthetic pathway showing trypanocidal activity have been described [21]. Since enzymes involved in the basic steps common to the biosynthesis of GPI in the different organisms have different sensitivities to various inhibitors [22], [23], [24], [25], [26], [27], we sought to characterize the genes involved in biosynthesis of GPI anchors in *T. cruzi*. Orthologous sequences of all genes involved in biosynthesis of *T. cruzi* GPI anchors were identified and, for three of them, we were able to show that they complement yeast conditional mutants of genes of this pathway. Unsuccessful attempts to generate *T. cruzi* knockouts for three of these genes suggest that GPI is an essential component of the parasite. Since specific inhibition of GPI biosynthesis may affect the expression of a large number of *T. cruzi* proteins that are essential for host-parasite interactions, targeting this pathway can be considered a promising strategy for the development of new chemotherapy against Chagas disease. The availability of yeast mutants expressing *T. cruzi* enzymes constitutes the first step in that direction.

## Methods

### Parasite cultures

Epimastigotes of the CL Brener clone of *T. cruzi* were maintained in logarithmic growth phase at 28°C in liver infusion tryptose (LIT) medium supplemented with 10% fetal bovine serum as described by Camargo [28]. Metacyclic trypomastigotes were obtained after metacyclogenesis in LIT medium, observed after 15–20 days of culture [28] and were used to infect Vero cells. Intracellular amastigotes and tissue culture derived trypomastigotes were obtained from Vero cells grown in Dulbecco's Modified Eagle Medium (DMEM) supplemented with 5% fetal bovine serum, at 37°C and 5% CO_2_ as previously described [29].

### *In silico* analysis of *T. cruzi* genes

Sequence analyses were conducted using the *T. cruzi* genome database (www.tritrypdb.org) to identify all orthologous genes involved in the parasite GPI biosynthesis. Sequences from different organisms, such as *T. brucei*, *P. falciparum* and *S. cerevisiae* [16], [20], were used as queries in Blastp analyses (www.ncbi.nlm.nih.gov/blast/Blast.cgi) and ClustalW (www.clustal.org/) for multiple alignments between the predicted *T. cruzi* protein sequences and homologous sequences present in other organisms.

### DNA and RNA extraction, northern blot and RT-PCR assays

Total DNA was purified from 10^9^ *T. cruzi* epimastigotes that were harvested from exponentially growing cultures, according to previously described protocols [29]. Total RNA was isolated from epimastigotes, tissue culture derived trypomastigotes and amastigotes using the RNeasy kit (Qiagen). For northern blot analyses, 10 µg of total RNA/lane was separated in 1.2% agarose/MOPS/formaldehyde gel. The RNA was transferred to Hybond-N membrane (GE-Healthcare) and hybridized with GPI8, GPI10 and 24Sα rRNA probes previously labeled with [α-^32^P]-dCTP using the Amersham Ready-to-Go DNA Labeling Beads (GE-Healthcare), according to the suppliers protocol. The hybridization was carried out as previously described [30] in 50% formamide buffer at 42°C. After washing twice with 2X SSC/0.2% SDS at 60°C for 20 min, the membranes were exposed to a phosphor screen of the STORM 820 phosphor image (GE-Healthcare). Reverse-transcription amplifications (RT-PCR) were carried out with total RNA isolated from transfected yeast mutants and *T. cruzi* epimastigotes according to published protocols [30]. After first strand cDNA synthesis using oligo (dT)_18_ or gene-specific primers (see primer sequences in supplementary material, Table S1) and the SuperScript II Reverse Transcriptase (Life Technologies), the cDNAs were amplified using Taq Polymerase (Promega) and primers specific for each gene and analyzed in 1% agarose gels stained with ethidium bromide.

### Yeast strains and culture media

The *S. cerevisiae* strain used in this work were: YPH499 (Mat a, *ura3-52*, *lys2-801amber*, *ade2-101ochre*, *trp1-63*, *his3-200*, *leu2-1*) (Stratagene), used as a control, and conditional lethal yeast mutants for GPI biosynthesis (YPH499-HIS-GAL-DPM1, YPH499-HIS-GAL-GPI3, YPH499-HIS-GAL-GPI8, YPH499-HIS-GAL-GPI10, YPH499-HIS-GAL-GPI12, YPH499-HIS-GAL-GPI14, YPH499-HIS-GAL-GAA1, and YPH499-HIS-GAL-AUR1), which were generated by replacement of the endogenous yeast promoter by a galactose regulated promoter, as described [31]. *S. cerevisiae* strains were grown in YPGR medium (1% w/v yeast extract, 2% w/v bacto-peptone, 2% w/v galactose, 1% w/v raffinose), or in SD medium (0.17% yeast nitrogen base, 0.5% ammonium sulfate, 2% glucose, containing the nutritional supplements necessary to complement the auxotrophic samples or to allow selection of transformants). Before complementation, yeast clones were cultivated in SGR medium (4% galactose, 2% raffinose, 0.17% yeast nitrogen base, 0.5% ammonium sulfate) in which glucose is replaced by galactose/raffinose as a carbon source.

### Transformation of conditional lethal *S. cerevisiae* mutants

Sequences encompassing the full-length coding regions of *TcDPM1*, *TcGPI3*, *TcGPI8*, *TcGPI10*, *TcGPI12*, *TcGPI14*, *TcGAA-1*, and *TcIPCS* were PCR amplified from total DNA of *T. cruzi* epimastigotes prepared as described above, using primers specific for each gene (Table S1). The amplicons were inserted into the *S. cerevisiae* expression vector pRS426Met [32]. Full-length coding sequences corresponding to orthologous *S. cerevisiae* genes were also PCR amplified with specific primers (Table S1) and cloned into the same vector. Transformation of yeast mutants were carried out using the standard lithium acetate procedure [33]. Conditional lethal mutants were transformed with pRS426Met plasmids carrying either the *S. cerevisiae* (Sc) or the *T. cruzi* (Tc) genes and transformed cells were plated on minimal medium lacking histidine and uracil containing either galactose (SGR) or glucose (SD) and incubated at 30°C.

### SDS-PAGE of [2-^3^H]*myo*-inositol labeled yeast proteins

Control YPH499 cells, mutant yeasts (YPH499-HIS-GAL) and mutant yeasts carrying pRS426Met containing yeast or *T. cruzi* genes were grown in SGR to saturation and used to inoculate SD (2% glucose), in which they were grown for about 16 h. Cells (1×10^8^) were washed twice in SD without inositol medium (2% glucose), resuspended in 1 ml of SD without inositol (2% glucose) and depleted of inositol for 20 min before the addition of 30 µCi of [2-^3^H]*myo*-inositol (American Radiolabeled Chemicals, St. Louis, USA). Cells were labeled for 1 hour. Protein extraction was done according to Damasceno et al. [34] with the following modifications: radiolabeled cells were harvested, washed twice in phosphate-buffered saline (PBS 1X) at pH 7.4, and resuspended in 100 µl of Yeast Breaking Buffer [50 mM sodium phosphate, pH 7.4; 1 mM phenylmethylsulfonyl fluoride (PMSF); 1X protease inhibitor cocktail (Amresco, Solon, USA); 1 mM EDTA, and 5% (v/v) glycerol]. Yeast cells were lysed by the addition of acid-washed glass beads (425–600 µm) vortexing for 1 min with 1 min intervals on ice, repeated twenty times. The lysate was centrifuged at 2,000×*g* for 5 min at 4°C and the supernatant was collected. The remaining pellet containing cell debris and glass beads was resuspended in 75 µl of Yeast Breaking Buffer containing 2% (w/v) sodium dodecyl sulfate (SDS) by vortexing for 1 min with 1 min intervals on ice, repeated five times. After removing cellular debris by centrifugation, the lysates were combined and the proteins were then separated by 10% SDS-polyacrylamide gel electrophoresis. Protein bands containing labeled inositol were detected by fluorography.

### Dol-P-Man synthase assays

Wild type and yeast mutant cell lysates were prepared as previously described [35]. Briefly, exponential-phase yeast cultures corresponding to 1.5×10^7^ cells/ml of cells grown in glucose-containing medium (nonpermissive) or in galactose-containing medium (permissive medium) were lysed after incubation in 1.0 ml of 1 M sorbitol/1 mM EDTA containing Zymolyase at 37°C and glass beads for 30 min, harvested by centrifugation (1800×*g*, 10 min, 4°C) and resuspended in 200 µl of TM buffer (50 mM Tris/HCl, pH 7.5, containing 5 mM MgCl_2_ and 0.2% 2-mercaptoethanol). Ninety µl for lysates (corresponding to 3×10^8^ cells for each assay) were assayed directly for Dol-P-Man synthase activity as described [36]. Briefly, incubation mixtures contained 5 µl of GDP-[^3^H]Man (1 µCi/ml), 1 µl of Dol-P (5 mg/ml dispersed in 1.0% Triton X-100 by sonication) and water to give a final volume of 10 µl. Amphomycin and tunicamycin (final concentrations 1 mg/ml) were added to some samples. After the addition of 90 µl of cell lysates and incubation at 30°C for 30 min, the reactions were terminated by the addition of 1.5 ml of ice-cold chloroform/methanol (2∶1, v/v). The reactions were centrifuged (1500×*g*, 5 min, 4°C) and the pellet extracted twice with 500 µl of chloroform/methanol. Equivalent amounts of radiolabeled, chloroform/methanol extractable reaction products were analyzed by TLC on Silica 60 plates (Merck) with chloroform/methanol/acetic acid/water (25∶15∶4∶2, by vol.) as solvent and Dol-P-Man as a reference. Plates were screened for radioactivity with a Berthold LB 2842 Automatic TLC-Linear Analyzer.

### Parasite transfections and cellular localization of GFP fusion proteins

Full-length *TcDPM1*, *TcGPI3*, and *TcGPI12* coding sequences were PCR amplified from genomic DNA purified from cultures of the *T. cruzi* epimastigotes, using forward and reverse primers carrying *Xba*I and *Eco*RI restriction sites, respectively (Table S1). The amplicons were inserted into the *Xba*I-*Eco*RI sites of the *T. cruzi* expression vector pTREXnGFP [37], generating pTREX-TcDPM1-GFP, pTREX-TcGPI3-GFP, and pTREX-TcGPI12-GFP that contain *TcDPM1*, *TcGPI3* and *TcGPI12* genes fused to the N-terminus of the green fluorescent protein (GFP). A total of 100 µg of each plasmid construction was used to transfect *T. cruzi* epimastigotes as previously described [37]. Twenty four hours post-transfection, parasites were fixed with 4% paraformaldehyde for 30 min at 4°C, permeabilized with 0.1% Triton X-100 for 5 min at room temperature and blocked with 5% fetal bovine serum in PBS (blocking solution) for 20 min at 4°C. Staining of the parasite ER was done with rabbit anti-*T. brucei* BiP antibody ([38]; kindly provided by Renato Mortara, Universidade Federal de São Paulo), at a 1∶1000 dilution, and secondary goat anti-rabbit IgG antibody conjugated to Alexa Fluor 555 (1∶1000 dilution) (Molecular Probes/Life Technologies). After nuclei staining with 1 µg/ml of 4′,6-diamidino-2-phenylindole (DAPI, Molecular Probes/Life Technologies), cover slides were mounted with 90% glycerol, 10% 1 M Tris HCl pH 9.0, and 2.3% DABCO (Sigma). Images were obtained with a fluorescence microscope (Nikon Eclipse Ti) or with the 5 LIVE confocal microscope (Zeiss), both at the Center of Electron Microscopy (CEMEL), at the Instituto de Ciências Biológicas, UFMG. Transfections of HT1080 human fibrosarcoma cells were done with 1 µg of pcDNA3.1/NT-GFP-TOPO (Life Technologies) containing the different *T. cruzi* genes inserted in fusion with GFP (for primer sequences, see Table S1) and the FuGENE transfection reagent (Roche), following the manufacturer's instructions. All plasmids were co-transfected with pGAG-DsRed-ER, a mammalian expression vector that encodes the *Discosoma* sp. red fluorescent protein (DsRed) in fusion with ER targeting sequences and the ER retention sequence, KDEL (Clontech).

### Disruption of *T. cruzi* genes

DNA constructs designed to delete both *TcGPI8* alleles in the *T. cruzi* CL Brener genome by homologous recombination were prepared after PCR amplification of the 5′ and 3′ regions of the *TcGPI8* gene (for primer sequences, see Table S1). The generated PCR products (with 487 bp and 647 bp, respectively) were cloned sequentially into the *Sac*I/*Spe*I and *Xho*I/*Xba*I sites of pCR2.1 TOPO vector (Invitrogen), flanking the neomycin phosphotransferase (*Neo^R^*) or hygromycin phosphotransferase (*Hyg^R^*) resistance markers that were cloned into this vector. To improve mRNA expression in the parasite, the 3′ UTR plus downstream intergenic sequences of the *T. cruzi* gliceraldehyde-3-phosphate dehydrogenase (*gapdh*) gene was inserted downstream from the *Hyg^R^* marker. Similar constructs using 5′ and 3′ flanking sequences derived from *TcGPI3* and *TcGPI10* genes were generated. Epimastigote transfections were performed by electroporation with 50 µg DNA as described previously [37]. Twenty-four hours after transfection, 200 µg/ml of hygromycin B or G418 was added to the cultures and selected populations were obtained approximately 30 days after transfection. Cloned cell lines were obtained by plating on semisolid blood agar plates, after another 30 days of incubation at 28°C.

### Electron microscopy analyses of *T. cruzi*

Epimastigotes were fixed in 5% glutaraldehyde in 0.1 M cacodylate buffer pH 7.2 and processed following standard protocols, including post-fixation in osmium tetroxide followed by block counterstained with uranyl acetate and embedding in Epon resin. Ultrathin sections were counterstaining with lead citrate and analyzed in the Transmission Electron Microscope Tecnai G2-12 - SpiritBiotwin FEI - 120 kV located at the Center of Microscopy at the Universidade Federal de Minas Gerais, Belo Horizonte, Brazil.

### Cell membrane preparation, immunoblot and flow cytometry analyses

Approximately 10^9^ epimastigotes were lysed in 20 mM Hepes, 10 mM KCl, 1.5 mM MgCl_2_, 250 mM sucrose, 1 mM DTT, 0.1 mM PMSF, with five cycles of freezing in liquid nitrogen and thawing at 37°C. Total cell lysate was centrifuged at a low speed (2,000×*g*) for 10 min and the supernatant was subjected to ultracentrifugation (100,000×*g*) for one hour. The resulting supernatant was analyzed as soluble, cytoplasmic fraction (C) whereas the pellet, corresponding to the membrane fraction (M) was resuspended in lysis buffer. Volumes corresponding to 20 µg of proteins from total parasite cell lysate (T), cytoplasmic (C) and membrane (M) fractions were loaded onto a 12.5% SDS-PAGE gel, transferred to nitrocellulose membranes, blocked with 5.0% non-fat dry milk and incubated with the anti-mucin antibody 2B10 (gently provided by Nobuko Yoshida, Universidade Federal de São Paulo), at 1∶200 dilution followed by incubation with peroxidase conjugated anti-mouse IgG and the ECL Plus reagent (GE-Healthcare). For flow cytometric analysis, epimastigotes were stained with anti-mucin 2B10 (dilution 1∶450) and Alexa Fluor 488 conjugated secondary antibodies. Data were acquired on a FACScan flow cytometer (Becton Dickinson).

## Results

### *In silico* identification of *T. cruzi* genes involved in the GPI biosynthetic pathway

**Figure 1:**
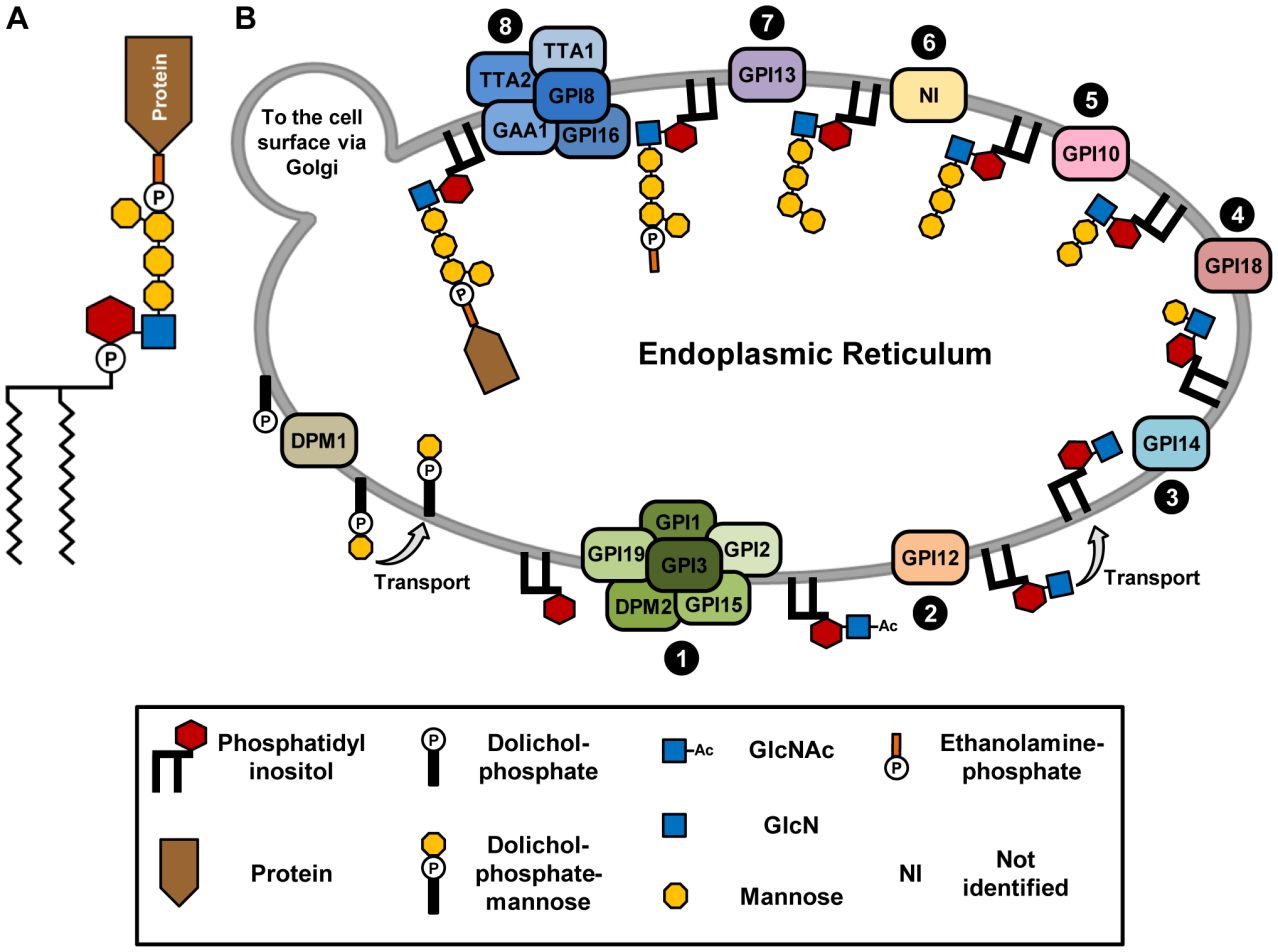
Structure and the biosynthesis of *T. cruzi* GPI anchors. (**A**) Structure of a *T. cruzi* GPI anchor, according to Previato et al. [3]. (**B**) Proposed biosynthetic pathway of GPI anchor in the endoplasmic reticulum of *T. cruzi*. N-acetylglucosamine (GlcNAc) is added to phosphatidylinositol (PI) in step 1 and, during the following steps, deacetylation and addition of four mannose residues occur. The addition of ethanolamine-phosphate on the third mannose (step 7) enables the transferring of the completed GPI anchor to the C-terminal of a protein (step 8). Dolichol-P-mannose acts as a mannose donor for all mannosylation reactions that are part of the GPI biosynthesis. This pathway was based on the structure of the *T. cruzi* GPI and sequence homology of *T. cruzi* genes with genes known to encode components of this pathway in *Saccharomyces cerevisiae*, *Homo sapiens*, *Trypanosoma brucei* and *Plasmodium falciparum*. Not shown in the figure, free glycoinositolphospholipids (GIPLs), also present in the *T. cruzi* membrane, are likely to be by-products of the same GPI biosynthetic pathway.

Eighteen *T. cruzi* genes involved in 8 steps of the GPI biosynthetic pathway were identified based on their similarities to the yeast, mammals, *Trypanosoma brucei* and *Plasmodium falciparum* sequences [15], [16], [17], [20], (Table 1). For the majority of these genes, annotated as putative *T. cruzi* orthologs in the TriTrypDB (www.tritrypdb.org), both alleles, belonging to the two CL Brener haplotypes, were identified. Since CL Brener is a hybrid strain, as described by El-Sayed et al. [39], the two haplotypes corresponding to the two ancestral genomes that originated the CL Brener genome, named Esmeraldo-like and non-Esmeraldo-like, were separated during the *T. cruzi* genome assembly. In Table 1, the genes corresponding to the non-Esmeraldo haplotype were indicated by their identification numbers in the TriTrypDB database. For all listed genes, the amino acid identities between the two alleles were greater than 94%. Based on these sequences and the known structure of the GPI anchor in this parasite (Figure 1A) [3], we proposed that the *T. cruzi* GPI biosynthetic pathway occurs in the ER according to the diagram shown in Figure 1B.

**Table 1:** *T. cruzi* genes encoding enzymes of the GPI biosynthetic pathway.

Step	*T. cruzi* gene	Gene ID (TriTrypDB*)	Number of amino acids	% Identity at protein level (**)	
				Yeast	Human
Dolicholphosphate-mannose synthase	*DPM1*	Tc00.1047053506581.10	260 aa	50% (DPM1)	31% (DPM1)
N-acetyl-glucosamine transferase (GlcNAc-PI) (Step 1)	*GPI1*	Tc00.1047053510329.200	827 aa	15% (GPI1)	16% (PIG-Q)
	*GPI2*	Tc00.1047053503781.20	336 aa	14% (GPI2)	32% (PIG-C)
	*GPI3*	Tc00.1047053509215.16	455 aa	41% (GPI3)	49% (PIG-A)
	*GPI15*	Tc00.1047053511655.10	307 aa	12% (GPI15)	18% (PIG-H)
	*GPI19*	Tc00.1047053508307.100	142 aa	10% (GPI19)	27% (PIG-P)
	*DPM2*	Tc00.1047053510043.29	100 aa	–	17% (DPM2)
GlcNAc-PI de-N-acetylase (Step 2)	*GPI12*	Tc00.1047053511481.40	252 aa	31% (GPI12)	32% (PIG-L)
α-1,4-Mannosyltransferase I (Step 3)	*GPI14*	Tc00.1047053511507.50	472 aa	30% (GPI14)	30% (PIG-M)
α-1,6-Mannosyltransferase II (Step 4)	*GPI18*	Tc00.1047053503521.89	529 aa	17% (GPI18)	20% (PIG-V)
α-1,2-Mannosyltransferase III (Step 5)	*GPI10*	Tc00.1047053510299.50	584 aa	21% (GPI10)	24% (PIG-B)
α-1,2-Mannosyltransferase IV (?) (Step 6)	ni	ni		– (SMP3)	– (PIG-Z)
Ethanolamine phosphotransferase (Step 7)	*GPI13*	Tc00.1047053503979.10	691 aa	15% (GPI13)	21% (PIG-O)
GPI transamidase (Step 8)	*GAA1*	Tc00.1047053504069.60	462 aa	10% (GAA1)	13% (GAA1)
	*GPI8*	Tc00.1047053511277.450	325 aa	31% (GPI8)	29% (PIG-K)
	*GPI16*	Tc00.1047053510877.180	684 aa	8% (GPI16)	9% (PIG-T)
	*TTA1*	Tc00.1047053510435.40	387 aa	–	–
	*TTA2*	Tc00.1047053508661.60	414 aa	–	–
GPI-inositol deacylase	*GPIdeAc2*	Tc00.1047053508153.1040	804 aa	9% (BST1)	12% (PGAP1)
Inositol phosphorylceramide synthase	*IPCS*	Tc00.1047053510729.290	335 aa	10% (AUR1)	–

(*) Gene ID numbers refer to the non-Esmeraldo-like haplotype, except for *TcGPI16* and *TcGPI19*, for which only the Esmeraldo-like alleles were identified.

(**) Names for the yeast and human orthologs are shown in parentheses.

ni: not identified.

Dolichol-phosphate mannose synthase (DPM1), also named dolichol-phosphate-β-D-mannosyltransferase, catalyses the transfer of a mannose residue from GDP-mannose to dolichol-phosphate (Dol-P) generating Dol-P-mannose, used as a donor for all mannosylation reactions that are part of the GPI biosynthetic pathway [40], [41]. Comparisons among DPM1 of various organisms [42], [43], [44] showed that, together with *S. cerevisiae*, *T. brucei*, and *Leishmania mexicana* [45] and in contrast to *P. falciparum* DPM1, *T. cruzi* DPM1 belongs to a group that includes monomeric enzymes that have a C-terminal hydrophobic tail. The glycosyltransferase complex that is responsible for transferring N-acetylglucosamine (GlcNAc) from UDP-GlcNAc to phosphatidylinositol (PI) to generate N-acetylglucosaminyl-PI (GlcNAc-PI) has six and seven proteins, respectively, in yeast and mammalian cells [16]. *TcGPI3* was identified as the gene encoding the catalytic subunit of the *T. cruzi* glycosyltransferase complex since it shares 41% and 49% of sequence identity with the yeast GPI3 and mammalian PIG-A, respectively. Among other components of the glycosyltransferase complex present in yeast, we identified the *T. cruzi* orthologs of GPI1, GPI2, GPI15, and GPI19. In mammalian cells, DPM2, a non-catalytic subunit of dolichol-P-mannose synthase, is physically associated with PIG-A, PIG-C and PIG-Q and enhances GlcNAc-PI transferase activity [46]. A *T. cruzi* gene encoding a protein with 17% identity to human DPM2 and containing a DPM2 domain, which probably acts as a regulatory component of the N-acetyl-glucosamine transferase complex, was also identified. Only one component of this complex, named ERI1 in yeast [47], and PIG-Y in mammals [48], was not identified either in *T. cruzi*, *P. falciparum* or *T. brucei*. The *T. cruzi* ortholog of yeast GPI12 (named PIG-L in mammals) [49], encoding the enzyme responsible for the de-N-acetylation of GlcNAc-PI, which has been well characterized in *T. brucei* [50], [51], was also identified. Since differences in substrate recognition among the mammal and *T. brucei* enzyme have been described [52], this enzyme has been considered as a suitable target for drug development.

As depicted in Figure 1B, the first two reactions of the GPI biosynthetic pathway occur on the cytoplasmic face of the ER, whereas mannosylation reactions occur in the ER lumen. After deacetylation, the GPI precursor is transported across the ER membrane to the ER lumen, a step that requires distinct flippases [53]. In yeast and mammalian cells, the addition of mannose residues to GlcN-PI after flipping this precursor into the ER lumen requires acylation of the inositol ring and, after mannosylation and the attachment of GPIs to proteins, this group is removed [54]. In contrast, in *T. brucei*, inositol acylation occurs after the addition of the first mannose residue [55] since both acylated and non-acylated GPI intermediates exist during transfer of the Man2 and Man3 to GPI intermediates [56]. Although analyses of GPI precursors synthesized in *T. cruzi* cell-free systems indicated that this organism also has the ability to acylate the inositol ring [57], sequences encoding an enzyme responsible for acylation of the inositol ring, named *PIG-W* in mammals and *GWT1* in yeast [54], [58] were not identified either in *T. cruzi* or in *T. brucei* [2]. In spite of that, the two alleles encoding the ortholog of the enzyme responsible for inositol deacylation, named GPIdeAc2 in *T. brucei* [56], were found in the *T. cruzi* genome (Tc00.1047053508153.1040 and Tc00.1047053506691.22).

All three genes encoding mannosyltransferases, responsible for the addition of the first, second and third mannose residues to GlcN-PI, named *TcGPI14* (α-1,4-mannosyltransferase), *TcGPI18* (α-1,6-mannosyltransferase) and *TcGPI10* (α-1,2-mannosyltransferase), were identified in the *T. cruzi* genome. Since the predicted *T. cruzi* proteins exhibit sequence identities with yeast and human proteins ranging from 17% to 30%, for some of these genes, functional assays are necessary to confirm these predictions. It is noteworthy that no *T. cruzi* ortholog encoding the enzyme responsible for the addition of the fourth residue of mannose (step 6), named SMP3 in yeast and PIG-Z in human, was identified. Similarly, no ortholog of the *SMP3* gene was found in *P. falciparum*, even though the presence of a fourth mannose residue has been shown by structural studies of the GPI anchor from both organisms [3], [20], [59]. Furthermore, genes encoding an essential component of the mannosyltransferase I complex named PBN1 in yeast and PIG-X in mammals, have not been identified either in *T. cruzi* or in *T. brucei* [60], [61].

In mammals and yeasts there are three enzymes that add ethanolamine-phosphate (EtNP) to different mannose residues: PIG-N/MCD4 (EtNP addition to Man1), PIG-G/GPI7 (Man2), and PIG-O/GPI13 (Man3) [2], resulting in the structure to which the protein will be linked. In *T. cruzi*, *T. brucei* and *P. falciparum*, EtNP addition occurs only at the third mannose [2], [20] and, as expected, only a *T. cruzi GPI13* ortholog was identified. However, it has also been shown in different *T. cruzi* strains, that GPI-linked proteins as well as free GIPLs have 2-aminoethylphosphonate (AEP) replacing EtNP at the third mannose residue and that an additional AEP is linked to GlcN in *T. cruzi* GPI anchors (for recent reviews, see [62], [63]).

After being assembled, the transfer of the GPI anchor to the C-terminal end of a protein is mediated by a transamidase complex that cleaves the GPI-attachment signal peptide of the nascent protein. In human and yeast, this complex consists of five ER membrane proteins, PIG-K/GPI8, PIG-T/GPI16, PIG-S/GPI17, PIG-U/GAB1 and GAA1 [64] in which GPI8 is considered the catalytic subunit [16], [65]. As shown in Table 1, we identified *T. cruzi GPI8*, *GAA1* and *GPI16* orthologs. Although orthologs of GPI17 and GAB1 were not identified in other trypanosomatids, genes encoding two other components of the transamidase complex, known as trypanosomatid transamidase 1 (TTA1) and TTA2, were also found in *T. cruzi* [66].

Besides differences in the glycan core, in *T. cruzi* GPI anchors, the phosphatidylinositol (PI) is replaced by inositolphosphorylceramide (IPC), a molecule also present in plants, fungi but not present in mammals [4]. This change in the lipid portion of the anchor occurs during the differentiation of epimastigotes into metacyclic trypomastigotes [67] and is observed in members of the large family of *trans*-sialidases [68]. Although it may not be considered part of the GPI biosynthetic pathway, the *T. cruzi* IPC synthase (*TcIPCS*) is thought to be a highly attractive drug target [69]. Based on that, Denny and collaborators [70] identified the ortholog of *AUR1*, that encodes the yeast IPC synthase [71], in *Leishmania major* and two closely related *T. cruzi* sequences encoding proteins sharing 52–53% identity with the *Leishmania* IPC synthase [70]. Our analysis confirmed that the two sequences described by Denny and collaborators [70] correspond to the two alleles of the *T. cruzi* IPC synthase (*TcIPCS*) gene present in the CL Brener genome, which are synthenic with the *L. major* and *T. brucei* orthologs.

### mRNA expression and subcellular localization analyses of *T. cruzi* enzymes

**Figure 2:**
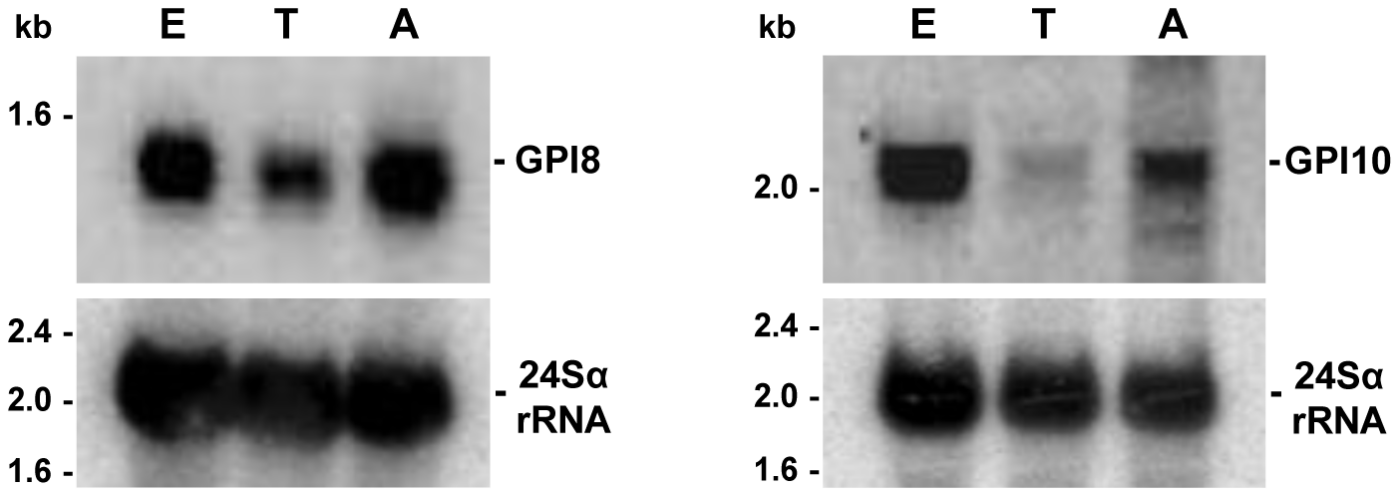
mRNA expression of *T. cruzi* genes encoding enzymes of the GPI biosynthetic pathway. Total RNA extracted from epimastigotes (E), trypomastigotes (T) and amastigotes (A) were separated in agarose gels, transferred to nylon membranes and hybridized with [α-^32^P]-labeled probes specific for *TcGPI8* and *TcGPI10* genes. The bottom panel shows hybridization with a probe for 24Sα rRNA, used as loading control. The size of ribosomal RNA bands are indicated on the left.

To verify whether the genes identified through the *in silico* analyses described above are expressed in *T. cruzi*, sequences encoding two enzymes of the GPI biosynthetic pathway were used as probes in northern blot hybridizations performed with total RNA purified from epimastigote, trypomastigote and amastigote forms of the parasite. As shown in Figure 2, transcripts with 1,300 nt and 2,100 nt, approximately, corresponding to *TcGPI8* and *TcGPI10* mRNAs were detected in all three stages of the parasite life cycle. As expected, increased levels of both transcripts were found in the two proliferative stages, epimastigotes and amastigotes, compared to the infective, nonproliferative trypomastigote stage.

**Figure 3:**
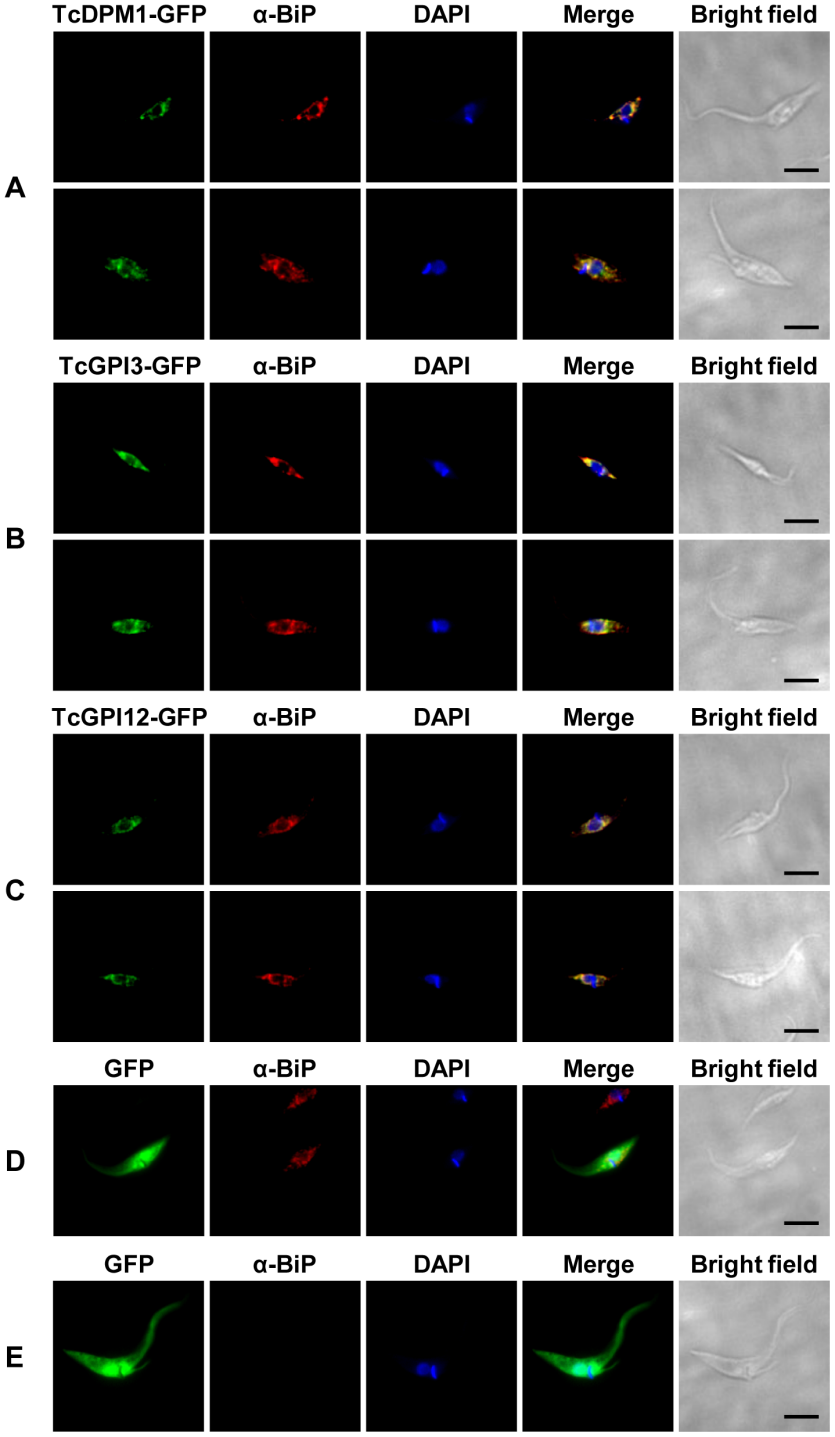
Cellular localization of *T. cruzi* enzymes of the GPI biosynthetic pathway. Epimastigotes were transiently transfected with the plasmids pTREX-TcDPM1-GFP (**A**), pTREX-TcGPI3-GFP (**B**), pTREX-TcGPI12-GFP (**C**) or pTREXnGFP as a control plasmid (**D**) and (**E**). Transfected parasites were fixed with 4% paraformaldehyde, incubated with the ER marker anti-BiP (1∶1000) and the secondary antibody conjugated to Alexa 555 (1∶1000). Cells were also stained with DAPI showing the nuclear and kinetoplast DNA. In panel **E**, parasites that were not incubated with the primary, anti-BiP antibody are shown as negative controls. Images were captured with the Nikon Eclipse Ti fluorescence microscope. Scale bars: 5 µm.

To provide further evidence for the role of the proteins encoded by the *T. cruzi* genes identified through *in silico* analyses as components of the GPI biosynthetic pathway, we determined the subcellular localization of three of these proteins expressed as GFP fusion in *T. cruzi* epimastigotes. The coding regions of *TcDPM1*, *TcGPI3* and *TcGPI12* genes were cloned in the *T. cruzi* expression vector pTREXnGFP and, after transfection into epimastigotes, the cells were examined by fluorescence microscopy. Figure 3 shows that all three fusion proteins in transfected parasites that were stained with anti-BiP antibodies [38] co-localize with BiP, a known ER marker. Similar results were obtained with confocal microscopy analyses (not shown), thus confirming that these enzymes are part of the GPI biosynthetic pathway. In addition, transfection of *T. cruzi* genes *TcDPM1*, *TcGPI3*, *TcGPI8* and *TcGPI12* in fusion with GFP in the HT1080 human fibrosarcoma cells also resulted in the expression of the GFP fusion *T. cruzi* proteins with a cellular localization compatible with the ER (Figure S1).

### Functional analyses of *T. cruzi* genes expressed in yeast

**Figure 4:**
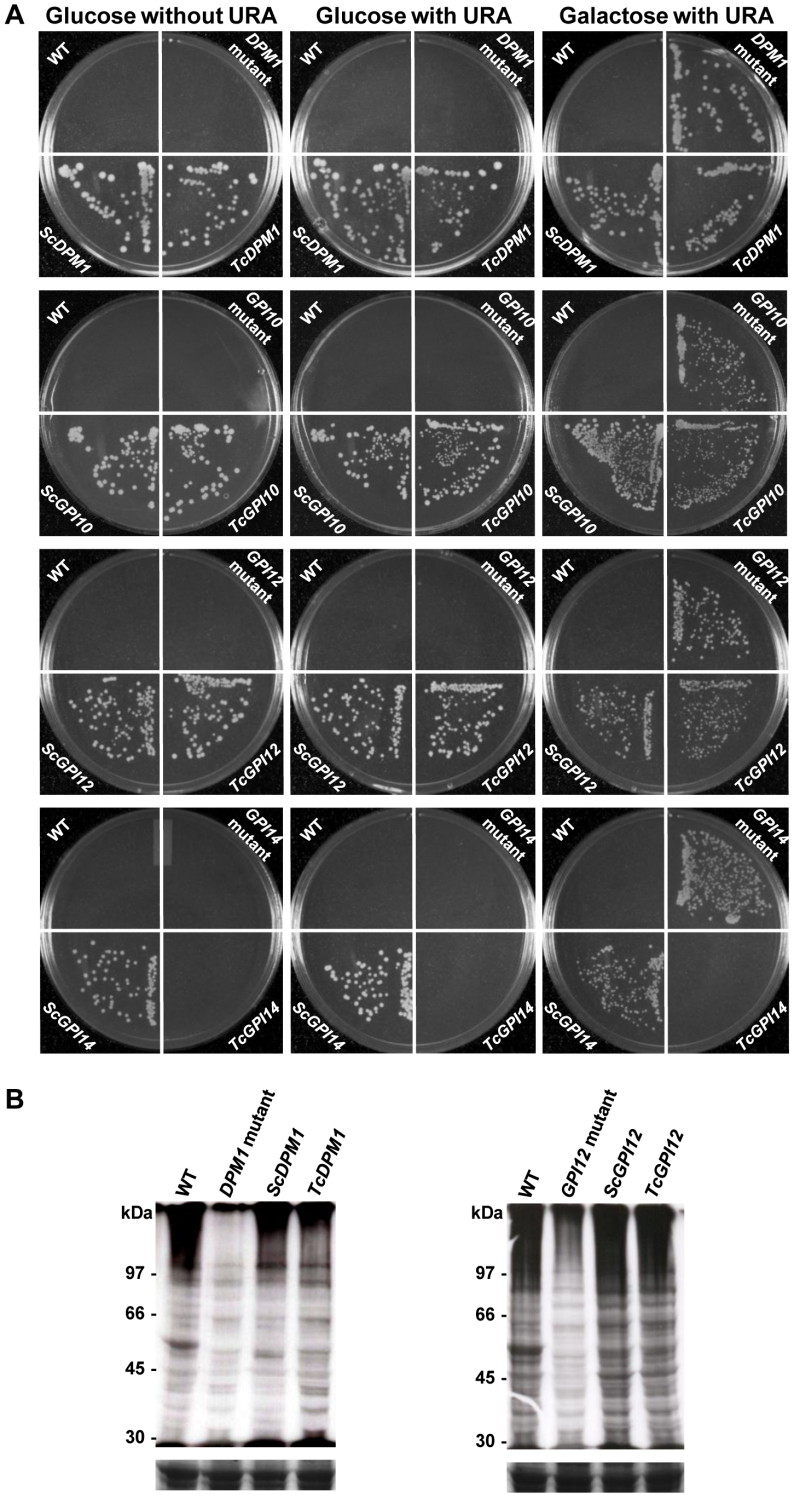
Yeast complementation with *T. cruzi* genes encoding enzymes of the GPI biosynthetic pathway. (**A**) *DPM1*, *GPI10* and *GPI12* yeast conditional lethal mutants (YPH499-HIS-GAL-DPM1, YPH499-HIS-GAL-GPI10 and YPH499-HIS-GAL-GPI12, respectively) were transformed with pRS426Met plasmids carrying either *T. cruzi* or *S. cerevisiae* genes encoding DPM1, GPI10 and GPI12 (*TcDPM1* or *ScDPM1*, *TcGPI10* or *ScGPI10*, and *TcGPI12* or *ScGPI12*, respectively). Wild-type (WT), non-transformed mutants and transformed yeast mutants were streaked onto plates with nonpermissive, glucose-containing SD medium lacking histidine, with or without uracil or in galactose-containing medium (with uracil) and incubated at 30°C for 3 days. In the bottom panel, yeast mutants (YPH499-HIS-GAL-GPI14) transformed with pRS426Met plasmid carrying *T. cruzi* gene (*TcGPI14*), which could not restore cell growth of GPI14 deficient yeast are shown. (**B**) GPI-anchored proteins synthesized by the conditional lethal yeast mutants expressing *T. cruzi* genes were separated by SDS-PAGE and analyzed after fluorography. Wild-type (WT), non-transformed yeast mutants and yeast mutants that were transformed with plasmids containing the corresponding yeast genes (*ScDPM1* or *ScGPI12*) or with the *T. cruzi* genes (*TcDPM1* or *TcGPI12*), were cultivated in medium glucose-containing in the presence of [2-^3^H]*myo*-inositol for 1 hour. Total protein extract corresponding to 1×10^8^ cells were loaded on each lane of a 10% SDS-PAGE and the labeled proteins were visualized by fluorography (top panels). As a loading control, Coomassie Blue stained gels prepared with equivalents amounts of total proteins are shown in the bottom panels. Untransfected *DPM1* and *GPI12* mutants were grown in the presence of galactose for 2 days and then switched to glucose-containing medium for 16 hours before addition of [2-^3^H]*myo*-inositol. Molecular weight markers (M) are shown on the left.

One of the main goals of this work is to develop a strategy for high-throughput screening of drugs against *T. cruzi* enzymes involved in the GPI biosynthetic pathway. *S. cerevisiae* has been largely used as surrogate system to express heterologous proteins from diverse parasites including *Leishmania* spp and *T. brucei*. Therefore, not only to assay for the functions of the *T. cruzi* genes but also to create yeast cells expressing *T. cruzi* target enzymes for future drug studies, conditional lethal yeast mutants were transformed with an expression vector containing the coding sequences for the *T. cruzi* genes *TcDPM1*, *TcGPI3*, *TcGPI12*, *TcGPI14*, *TcGPI10*, *TcGAA1*, *TcGPI8* as well as with the *TcIPCS*. These mutants were constructed by replacing the endogenous promoter of each one of the GPI genes by the GAL 1 promoter, resulting in yeast cell lines that could only grow in the presence of galactose [31]. By inhibiting the expression of the endogenous GPI genes in medium containing glucose, the complementation of yeast cells with the *T. cruzi* genes can be easily accessed by comparing the growth of transformed colonies in glucose and galactose-containing medium. As shown in Figure 4A and Table 2, we tested eight *T. cruzi* genes for which yeast mutants were available. Three of them, *TcDPM1*, *TcGPI10* and *TcGPI12*, once transformed into yeast, allowed the yeast mutants to grow on plates containing glucose as well as galactose. For all tested yeast mutants, we verified that transformation with plasmids containing the orthologous yeast gene allows them to grow on glucose-containing medium. Figure 4A also shows that, when the mutants were plated on glucose-containing medium supplemented with uracil, none of them were able to grow. As expected, wild type yeast, which has histidine deficiency, does not grow in minimum media lacking histidine. As an additional control, we verified, by RT-PCR analyses, the expression of two *T. cruzi* genes transformed into yeast mutants, for which we did not observed the complementation, i.e., that did not grow in nonpermissive media. Transcripts derived from the *T. cruzi TcGPI8* or *TcIPCS* genes, as well as from the orthologous yeast genes, were detected in the corresponding yeast mutants growing in galactose-containing media (Figure S2), indicating that the inability of these mutants to grow in the presence of glucose is not due to the lack of expression of the *T. cruzi* genes in the transfected yeasts.

**Table 2:** Functional complementation of yeast mutants by *T. cruzi* genes.

Yeast mutants	pRS Tc
YPH499 DPM1	+
YPH499 GPI3	−
YPH499 GPI12	+
YPH499 GPI14	−
YPH499 GPI10	+
YPH499 GAA1	−
YPH499 GPI8	−
YPH499 AUR1	−

The (+) signs indicate the ability of transformed mutants to grow in nonpermissive glucose-containing media.

To evaluate whether the expression of *T. cruzi* enzymes in yeast results in the correct synthesis of GPI anchor precursors by the complemented mutants, SDS-PAGE and fluorography analyses of yeast proteins containing [2-^3^H]*myo*-inositol were performed. As shown in Figure 4B, after 1 hour growing in medium containing glucose and [2-^3^H]*myo*-inositol, a complex pattern of proteins is visualized by fluorography in wild type cells as well as in yeast mutants expressing the *T. cruzi* genes. The protein patterns in yeast mutants expressing *TcDPM1* and *TcGPI12* genes growing in glucose-containing medium were indeed indistinguishable from the pattern observed with molecules synthesized by wild type yeasts or by mutants transformed with the orthologous yeast genes. On the other hand, a much weaker signal was detected in non-transformed yeast mutants, indicating that the expression of *T. cruzi* orthologs encoding enzymes of the GPI biosynthetic pathway restores the mutants' ability to synthesize GPI molecules.

Corroborating the functional complementation of yeast mutants with the *TcDPM1* gene, thin layer chromatography (TLC) of yeast mutants expressing the *T. cruzi* gene or the yeast *ScDPM1*gene, as a positive control, showed the presence of dolichol-P-mannose. Yeast cell extracts were preincubated with dolichol-phosphate and labeled *in vitro* with GDP-[2-^3^H]mannose. Labeled dolichol-P-mannose was detected in wild type yeast cells as well as in *DPM1* mutants that were transfected with the *TcDPM1* or with the yeast *ScDPM1* gene, confirming that the expression of the *T. cruzi* enzyme rescues the mutant ability to synthesize dolichol-P-mannose (Figure S3).

### *T. cruzi* GPI8 mutants have altered cell surface

**Figure 5:**
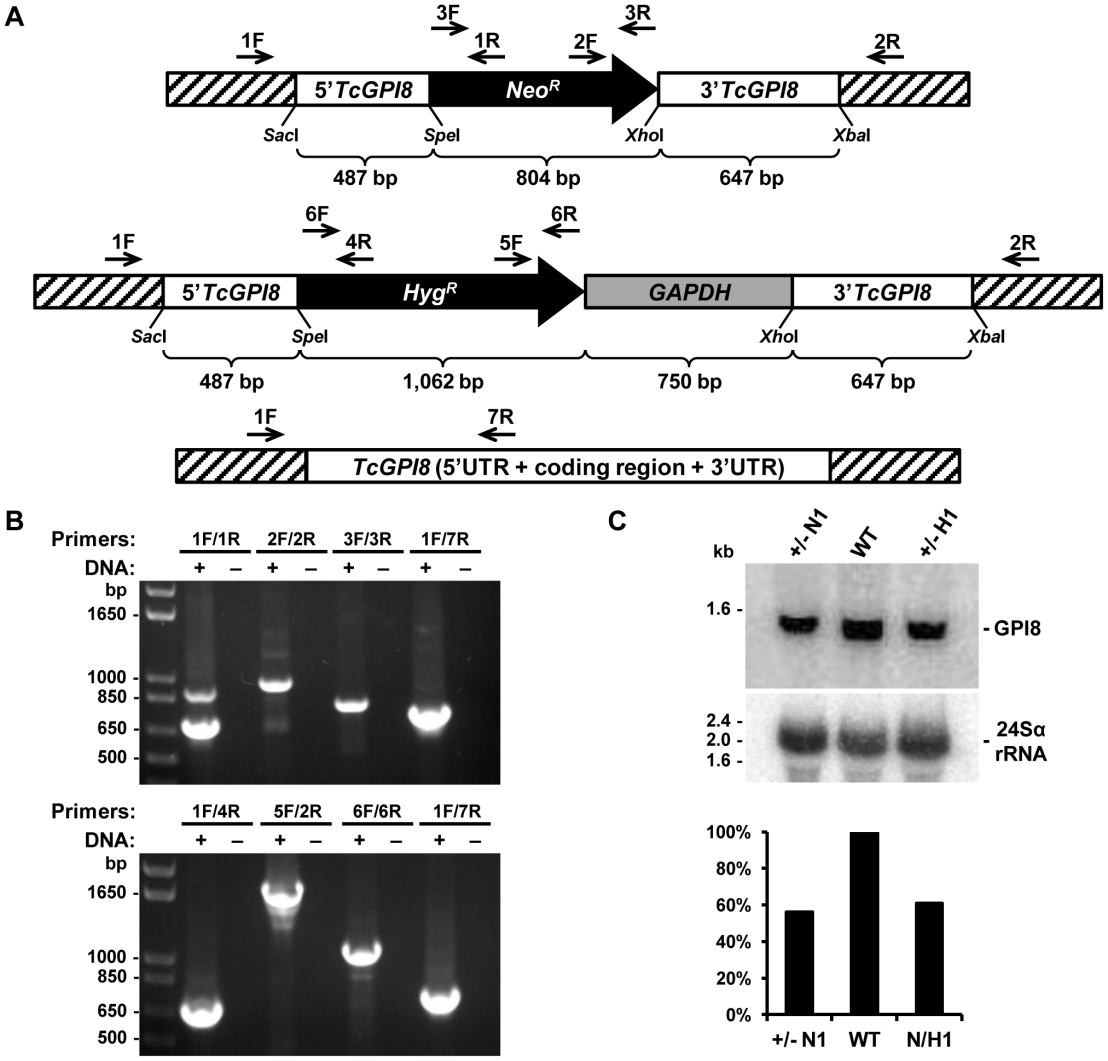
Generation of *TcGPI8* heterozygous mutants. (**A**) DNA constructs generated to delete both *TcGPI8* alleles by homologous recombination are shown with the *Neo^R^* or *Hyg^R^* genes flanked by 5′ and 3′ sequences of the *TcGPI8* gene and the *Sac*I/*Spe*I and *Xho*I/*Xba*I cloning sites from the pCR2.1TOPO vector. After transfecting epimastigotes with the purified DNA fragments, parasites were selected in LIT medium containing 200 µg/ml of G418 or hygromycin. Total DNA, isolated from G418 or hygromycin resistant parasites was analyzed by PCR amplifications, using the primers indicated by arrows. Below the schemes of DNA constructs, the sizes of the *Neo^R^* or *Hyg^R^* genes and the 5′ and 3′ sequences of the *TcGPI8* gene are shown. (**B**) PCR amplification products analyzed on 1% agarose gel electrophoresis were obtained from DNA isolated from epimastigotes transfected with the GPI8-Neo (top panel) or GPI8-Hyg construct (bottom panel) and using pairs of primers showed in A. Amplicons derived from PCR using the primer pair 1F/7R that amplify a *T. cruzi GPI8* allele which was not deleted are shown. On lanes indicated by (-), loaded samples were from PCR in which no template DNA was added. (**C**) Expression levels of *TcGPI8* mRNA in WT and *TcGPI8* single knockout of each allele interrupted by *Neo^R^* or *Hyg^R^* genes (+/− N or +/− H, respectively). Total RNAs purified from epimastigotes were hybridized to [α-^32^P]-labeled probes specific for the *TcGPI8* gene (top panel) or for the 24Sα rRNA (bottom panel) used as loading control. The size of ribosomal RNA bands are indicated on the left and a graph with the quantification of the signals from the *TcGPI8* probe after normalization using the 24Sα rRNA probe is shown below.

**Figure 6:**
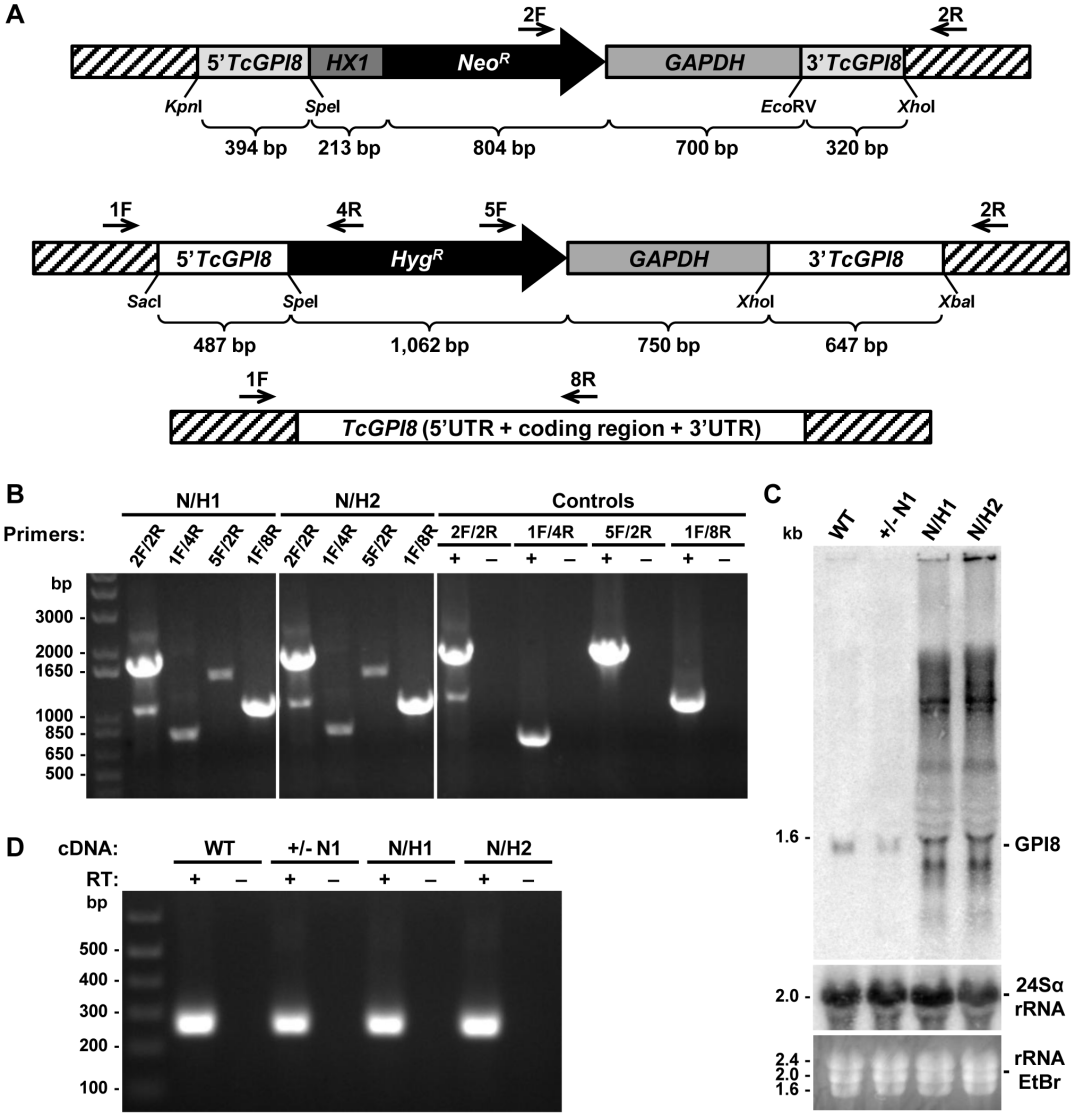
Translocation of the *TcGPI8* gene in *T. cruzi* mutants. (**A**) DNA constructs generated to delete both *TcGPI8* alleles are shown with the *Neo^R^* or *Hyg^R^* genes flanked by 5′ and 3′ sequences of the *TcGPI8* and spliced leader (SL) addition and polyadenylation signals from TcP2β (HX1) and *gapdh* genes. (**B**) DNA isolated from two cloned epimastigote cell lines that have been sequentially transfected with *Neo^R^* and *Hyg^R^* constructs and selected with G418 and hygromycin (double resistant mutants, N/H) were PCR amplified with primers shown in (A). Amplicons generated using primers 2F/2R indicate the integration of the *Neo^R^* in one of the *TcGPI8* alleles whereas amplicons generated with primers 1F/4R and 5F/2R indicate the integration of the *Hyg^R^* sequences in the second *TcGPI8* allele. PCR amplification using primers 1F/8R shows that double resistant parasite cell lines still maintained at least one intact copy of the *TcGPI8* gene. As positive controls, DNA from *Neo^R^* (for primers 2F/2R), *Hyg^R^* (for primers 1F/4R and 5F/2R) single knockout and wild-type parasites (for primers 1F/8R) were used. (**C**) Expression levels of *TcGPI8* mRNA in WT, *TcGPI8* single knockout *Neo^R^* (+/− N1) and two double resistant clones (N/H1 and N/H2). RNA purified from epimastigotes were hybridized to [α-^32^P]-labeled *TcGPI8* (top panel) or 24Sα rRNA (middle panel) probes. (**D**) RT-PCR amplification of *TcGPI8* sequences. Reverse transcribed TcGPI8 mRNA obtained from WT, single knockout *Neo^R^* (+/− N1) and two double resistant clones (N/H1 and N/H2) were PCR amplified with primers annealing with *TcGPI8* sequences and the *T. cruzi* SL sequence. First strand cDNA synthesis reactions were done with primers complementary to *TcGPI8* in the presence (+) or absence (−) of reverse transcriptase. PCR products, separated on 1% agarose gels were stained with ethidium bromide. Molecular weight DNA markers are shown on the left.

**Figure 7:**
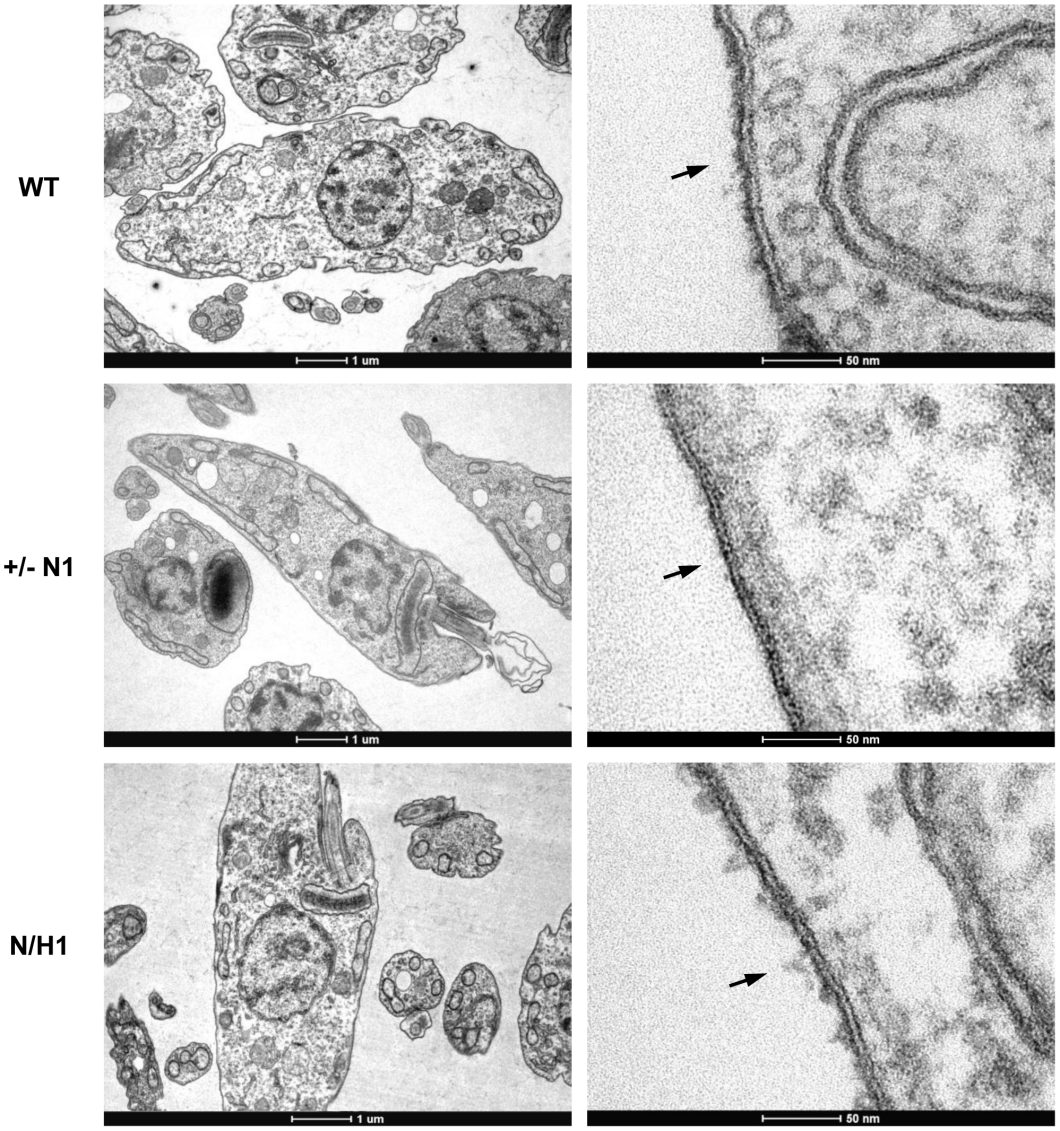
Cell membrane morphology of *T. cruzi GPI8* mutants. Transmission electron microscopy showing cellular membranes of wild type *T. cruzi* epimastigotes (WT), *TcGPI8* single allele knockout, neomycin resistant (+/− N1) and double resistant *TcGPI8* epimastigote mutants (N/H1). Although displaying similar morphologies, representative images show that single allele *TcGPI8* mutants present a thinner layer of parasite glycocalyx, when compared to wild type cells, whereas cell membranes of double resistant parasites present a glycocalyx layer that is slightly thicker than the glycocalyx of wild type parasite membranes (indicated by the arrows).

**Figure 8:**
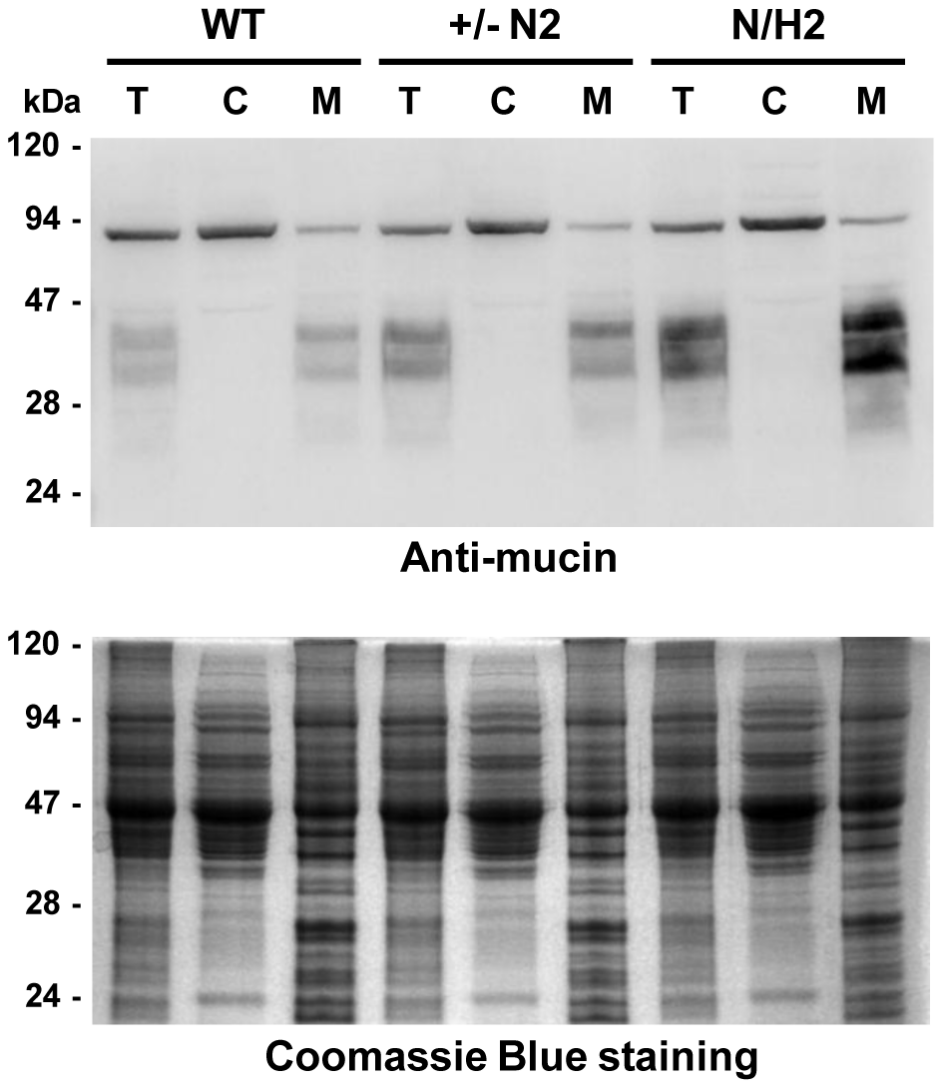
Cell membrane mucins in *T. cruzi GPI8* mutants. Immunoblot of total (T), cytoplasmic (C) and membrane (M) fractions of WT epimastigotes, *TcGPI8* single allele knockout, neomycin resistant (+/−N2) and double resistant *TcGPI8* (N/H2) mutant cell lines. Equivalent amounts of protein from each fraction, as showed by the Coomassie blue stained bands (bottom panel), were transferred to nitrocellulose membranes and incubated with anti-mucin antibodies and revealed with horseradish peroxidase conjugated secondary antibodies.

Knockout parasites of *GPI8*, *GPI16* and *GPI10* were generated in *T. brucei* whereas a *GPI8* knockout was described in *L. mexicana* [18], [19], [72], [73]. To further investigate the role of GPI anchors in *T. cruzi*, we tried to generate parasite cell lines in which both alleles of *TcGPI3*, *TcGPI8* and *TcGPI10* genes were deleted by homologous recombination. Although we were able to generate heterozygote epimastigotes carrying a drug resistance marker inserted in each one of the *TcGPI8* alleles (Figure 5A–B), several attempts to generate double-resistant, null mutant epimastigotes with both *TcGPI8* alleles deleted were unsuccessful. Also unexpectedly, transfection with plasmid constructs containing *TcGPI3* and *TcGPI10* sequences flanking the neomycin resistance gene did not result in G418 resistant parasites, indicating that disruption of even one allele of a gene involved in the initial steps of the GPI biosynthesis pathway results in non-viable parasites (not shown). Thus, our results suggest that, in contrast to *T. brucei* and *L. mexicana*, the GPI biosynthesis may be an essential pathway in epimastigotes of *T. cruzi*. In agreement with PCR analyses that showed the disruption of single alleles of *TcGPI8* (Figure 5B), northern blot assays (Figure 5C) showed that both heterozygous *TcGPI8* mutants have the expression of *TcGPI8* mRNA reduced by about 40%. Although a few double-resistant epimastigote clones were generated and PCR analyses indicated that the neomycin and hygromycin resistance genes were inserted into both *TcGPI8* alleles, PCR amplifications also indicated that additional sequences corresponding to the *TcGPI8* gene were present in a different genomic location in the double resistant parasites (Figure 6A–B). It should be noted that it was possible to generate the double resistant parasites only after we prepared different plasmid constructs in which the resistance genes were linked to trans-splicing and polyadenylation signals from the glyceraldehyde-3-phosphate dehydrogenase (*gapdh*) and the ribosomal protein TcP2β (*HX1*) genes and performing drug selection by gradually increasing drug concentrations. Northern blot analyses (Figure 6C) indicate that the recombination events that resulted in viable, double resistant parasites allowed the expression of an aberrant *TcGPI8* mRNA population. Among this *TcGPI8* mRNA population transcribed in the double resistant mutants, mature, trans-spliced mRNAs were detected by RT-PCR using primers specific for *TcGPI8* sequences and the *T. cruzi* spliced leader (Figure 6D), thus indicating that this gene is still active in these mutants. Although no significant changes in either growing or overall morphology of the *TcGPI8* mutants were observed, transmission electron microscopy showed striking alterations in the dense glycocalyx that covers the parasite surface. As shown in Figure 7, cell membranes of epimastigotes from *TcGPI8* heterozygous mutants (+/−N) present a thinner layer of the surface glycocalyx compared to wild type (WT) epimastigotes. In contrast, cell membranes from both clones of double resistant parasites (N/H), which may have suffered recombination events involving *TcGPI8* sequences, present an increased thickness of their glycocalyx compared to the heterozygous mutants (Figure 7). Although no significant differences in the levels of mucins were detected in the heterozygous mutants, western blot analyses of membrane proteins of WT and double resistant *TcGPI8* mutants using the anti-mucin monoclonal antibody 2B10 [74] showed increased amounts of the 35–50 kDa glycoproteins (also known as Gp35/50 mucins) expressed on the surface of epimastigotes of the double resistant clones (Figure 8). Flow cytometry of epimastigotes stained with 2B10 antibodies also showed increased amounts of surface mucins in the double resistant parasites (Figure S4).

## Discussion

Several *T. cruzi* surface proteins known to be involved in parasite infectivity or escape from the host immune response are anchored to the parasite membrane by covalent linkage to glycosylphosphatidylinositol (GPI). *T. cruzi* GPI anchors are also strong proinflammatory molecules, being critical in the modulation of the host immune response against this parasite. *T. cruzi* belongs to a group of early branching eukaryotic protists that are responsible for several neglected human tropical diseases, for which there is a strong need for new drug treatments. The elucidation of the structures of molecules that play a direct role in host-parasite interactions and the understanding of the biosynthetic pathways that generate these specific parasite molecules, such as the *T. cruzi* GPI biosynthetic pathway, represent a significant contribution towards this goal. Using a combination of sequence similarity analyses based on yeast, mammal, *T. brucei* and *P. falciparum* previously characterized genes, cellular localization and functional expression in yeast mutants we identified 18 orthologous genes encoding components of the GPI biosynthetic pathway present in the published *T. cruzi* CL Brener genome database. In addition, the gene encoding the IPC synthase, an enzyme responsible for a key modification that occurs in the lipid anchor during in the infective, trypomastigote stage of the parasite, was also identified. Although most sequences were correctly annotated in the TriTrypDB genome database, *TcGPI15*, *TcGPI19*, *TcDPM2* and *TcGPI16* were not correctly identified. It should be noted however that, because the assembly of the CL Brener genome is not complete, our *in silico* analyses might still contain a few missing genes whose presence or absence can only be unambiguously determined by a total sequencing read based analysis or through degenerate PCR experiments. Efforts towards identifying additional genes, such as the ones encoding a component of the mannosyltransferase I complex, α-1,2-mannosyltransferase IV or the acyltransferase, responsible for acylation of the inositol ring, are underway.

By expressing these genes in yeast mutants, we generated yeast cell lines that can now be used in high throughput screening assays for drugs that are specifically targeted to *T. cruzi* enzymes. Using yeast as a tool for drug screening against parasites is a strategy that has been successfully employed [5], [21], [75]. This system allows the identification of drugs acting specifically on the parasite enzyme since their effect on transfected yeast mutants growing in permissive and nonpermissive media can be compared (for a recent review, see [76]). Alternatively, specific inhibitors can be discovered using cell-free system assays, as it was shown for *T. brucei* and *L. major* enzymes involved with GlcNAc-PI de-N-acetylation, mannosylation and inositol acylation [23], [24], [25]. It is noteworthy that, among all tested genes, we observed functional complementation in yeast only for those whose products are not part of a protein complex. Among the *T. cruzi* genes that we were able to show complementation is the *DPM1* gene. Since all four mannose residues are likely to be transferred from dolichol-P-mannose, *DPM1*, a gene encoding the dolichol-P-mannose synthase is considered an excellent candidate gene to be targeted for drug test studies. In contrast to DPM1, for which the *T. cruzi* homologous protein has high levels of amino acid identity with the yeast enzyme, TcGPI10 was also able to complement the yeast mutation even though it has only 21% identity with the yeast enzyme. On the other hand, the *T. cruzi* IPC synthase, which presents 10% identity with the yeast enzyme and is also a promising target for chemotherapy against trypanosomiases, is not functional in yeast. This is an unexpected result, since it has been shown that the *Leishmania major* IPC synthase gene (also known as *AUR1* gene) restored the growth of yeast *AUR1* mutants in nonpermissive, glucose-containing media [70].

We further confirmed the role of these genes by analyzing the cellular localization and mRNA expression of their gene products. Sequences corresponding to *TcDPM1*, *TcGPI3* and *TcGPI12* genes, expressed as GFP-fusion proteins in epimastigotes, showed a cellular localization compatible with ER. Interestingly, the *T. cruzi* sequences containing ER localization signals can be recognized by the mammalian protein trafficking machinery, since we were also able to show similar localization of GFP fusions of TcDPM1, TcGPI3, TcGPI8 and TcGPI12 in the ER of transfected HT1080 human fibrosarcoma cells. As expected, analyses of mRNA levels of *TcGPI8* and *TcGPI10* indicated that the components of the GPI biosynthetic pathway are more actively produced in the two proliferative stages of the parasite life cycle, epimastigotes and amastigotes.

To gain further insights into the role of GPI molecules as well as GPI-anchored proteins, we tried to generate *T. cruzi* null mutants for some of these genes. Because a large number of *T. cruzi* proteins involved in host-parasite interactions such as members of the large *trans*-sialidase, mucin and MASP families are GPI anchored, the availability of *T. cruzi* cell lines with disrupted genes of the GPI biosynthetic pathway would allow us to perform a number of studies regarding the effect of the absence of these proteins on the parasite surface during infection. Given that it encodes the catalytic subunit of the GPI:protein transamidase complex, responsible for transferring GPI anchor to the proteins, we sought to disrupt the *TcGPI8* gene, which would have resulted in parasites containing only surface GIPLs, but no GPI-anchored proteins. Not surprisingly, the deletion of a single *TcGPI8* allele could be easily achieved by homologous recombination between sequences from each allele flanking the neomycin or hygromycin resistance genes. Accordingly, mRNA expression analyses showed that both *TcGPI8* heterozygous mutants have decreased mRNA levels. On the other hand, several attempts to delete the second *TcGPI8* allele did not result in viable parasites. When the plasmid constructs were modified and drug selection protocol was conducted in such a way that drug concentrations were increased gradually, rare double resistant cell lines were obtained. However, these parasites seem to have undergone large gene rearrangement involving *GPI8* sequences. Although frequently described in *Leishmania* spp, where gene amplification and overexpression of sequences have been observed after disruption of essential genes [45], [77], this phenomenon has been rarely reported for *T. cruzi* [78]. Together with the results of northern blot and RT-PCR analyses, preliminary data on pulse field gel electrophoresis and southern blot hybridizations (not shown) suggested that the amplification of *TcGPI8* sequences involved the production of episomal DNA molecules. Thus, the anomalous expression of *TcGPI8* mRNA sequences from distinct genomic locations, indicated by a large smear of high molecular weight RNA bands in northern blots and the amplification of spliced leader containing *TcGPI8* mRNA allowed the growth of mutants in which both *TcGPI8* alleles were disrupted by drug resistance markers. Surprisingly, although no major morphological alterations were evident, electron microscopy analyses of cell membrane structures of epimastigotes showed that *TcGPI8* mutants have changes in their glycocalyx layer. Even though the small reduction in the glycocalyx layer observed in the heterozygous mutants could not be correlated with changes in the levels of mucins, western blot with membrane fractions, confirmed by flow cytometry using anti-mucin antibodies indicated that double-resistant parasites present a small increase in the amount of surface glycoproteins, most likely due to an increased expression of the translocated copies of *TcGPI8* gene. Mucins play a critical role during infection, since they are the acceptors of sialic acid that allows trypomastigotes to build a negatively charged coat that protects them from killing by host anti-α-galactopyranosyl antibodies [79]. Whether the genomic rearrangements that resulted in the expression of *TcGPI8* from different genomic locations have affected the expression of other *T. cruzi* genes, it remains to be determined. It will be also important to determine which are the mechanisms employed by the parasite that resulted in the genomic rearrangement observed with the double resistant clones.

Interestingly, despite being viable in culture, *T. brucei* mutants lacking *TbGPI8* resulted in the absence of GPI-anchored surface proteins, accumulation of non-protein-linked GPI and incapacity of procyclic forms to establish infections in the tsetse midgut [80]. In contrast, *GPI8* RNAi knock-down in bloodstream forms resulted in accumulation of unanchored variant surface glycoprotein (VSG) and cell death with a phenotype indicative of blocking cytokinesis [72]. On the other hand, *L. mexicana GPI8* knockouts, although deficient of GPI-anchored proteins, display normal growth in culture, are capable of differentiating into amastigotes, and are able to infect mice [19]. In addition to *GPI8*, procyclic *T. brucei* lacking the *TbGPI12* and *TbGPI10* were also obtained. Although unable to synthesize GPI structures beyond GlcNAc-PI, *TbGPI12^−/−^* parasites are viable in culture, but are not able to colonize the tsetse midgut [51]. Deletion of *TbGPI10* also interferes with the ability of procyclic mutants to infect tsetse flies [18]. These reports are in contrast with our results indicating that disruption of only one allele of a gene involved in the initial steps of the GPI pathway such as *TcGPI3* or *TcGPI10* resulted in non-viable *T. cruzi* epimastigotes. On the other hand, similarly to the genomic alterations we observed in the *T. cruzi* double resistant *TcGPI8* mutants, an attempt to create a *L. mexicana* knockout by targeted deletion of the gene encoding the dolichol-phosphate-mannose synthase resulted in amplification of this chromosomal locus [45]. Thus, our contrasting results attempting to generate *T. cruzi* null mutants of genes involved with GPI biosynthesis, compared to similar studies described in *T. brucei* and *L. mexicana*, suggest that, although considered closely related organisms, the different members of the trypanosomatid family have significant peculiarities that deserve detailed analyses of major biochemical pathways in each parasite species.

## Supporting Information

Figure S1 **Cellular localization of** ***T. cruzi*** **proteins expressed in mammalian cells.** The *T. cruzi* genes *TcDPM1*, *TcGPI3*, *TcGPI12*, and *TcGPI8* were cloned in fusion with GFP in the vector pcDNA3.1/NT-GFP-TOPO and transfected into HT1080 human fibrosarcoma cells. Forty eight hours after transfections with pcDNA-GFP-TcDPM1 (**A**), pcDNA-GFP-TcGPI3 (**B**), pcDNA-GFP-TcGPI12 (**C**), pcDNA-GFP-TcGPI8 (**D**) or after mock transfections (**E**), cells were stained with DAPI and visualized under fluorescence microscopy. All plasmids were cotransfected with the pGAG-DsRed-ER plasmid to visualize cellular ER compartments. Scale bars: 20 µm.
(TIF)

Figure S2 **RT-PCR mRNA analysis of yeast mutants transformed with** ***T. cruzi*** **genes.** Reverse-transcription and PCR amplifications (RT-PCR) of total RNA isolated from non-transformed yeast mutants or mutants transformed with *T. cruzi* genes were analyzed by agarose gel electrophoresis. Total RNA was isolated from *GPI8* yeast mutants (top panel) or *AUR1* mutants (bottom panel). mRNA expression was analyzed in non-transformed mutants (*GPI8* mutants or *AUR1* mutants) or mutants transformed with pRS426Met plasmids carrying either the *T. cruzi* (*TcGPI8* or *TcIPCS*) that were grown in galactose-containing media. For each RNA sample, pair of primers used for cDNA amplifications, which are specific for the *TcGPI8*, *TcIPCS*, the endogenous *ScGPI8* or *ScAUR1*, as well as for the yeast *26S rRNA* genes, are indicated above each lane of the gel and are listed in Table S1. It is also indicated above each lane, whether the amplicons were generated in presence (+) or in the absence (−) of reverse transcriptase (RT). Molecular weight DNA markers are shown on the left.
(TIF)

Figure S3 **Synthesis of dolichol-P-mannose in yeast mutants expressing the** ***TcDMP1*** **gene.** Thin Layer Chromatography (TLC) of dolichol-phosphate-mannose *in vitro* labeled with GDP-[2-^3^H]mannose was performed using membrane fractions from: wild type yeast expressing the *DPM1* endogenous gene (**A**), grown in the complete medium and preincubated with dolichol-phosphate; (**B**) *DPM1* mutant grown in SD medium supplemented with uracil (nonpermissive conditions); (**C**) wild type yeast, expressing the *DPM1* endogenous gene, grown in the YPGR medium and preincubated with amphomycin and dolichol-phosphate; (**D**) *DPM1* mutant transformed with the recombinant plasmid pRS426Met containing the *ScDPM1* grown in nonpermissive medium; (**E**) WT yeast, containing the *DPM1* endogenous gene, grown in complete but not preincubated with amphomycin and dolichol-phosphate; (**F**) *DPM1* mutant transformed with the recombinant plasmid pRS426Met containing the *TcDPM1* grown in nonpermissive medium. The position of the dolichol-P-mannose (Dol-P-Man) in the TLC is indicated by an arrow.
(TIF)

Figure S4 **Flow cytometry analyses of** ***T. cruzi*** **mutants.** Wild type epimastigotes (WT), two *TcGPI8* single knockouts *Neo^R^* (+/− N1 and +/− N2) and double resistant clones (N/H1 and N/H2) were stained with the anti-mucin monoclonal antibody 2B10 (dilution 1∶450) and analyzed by flow cytometry. The values of mean fluorescence intensity (MFI) for each parasite cell line are shown below.
(TIF)

Table S1 **Sequences of oligonucleotides used for PCR amplications and to generate plasmid constructs.**
(PDF)
