# Supplementary figures and images for: Identification and Functional Analysis of Trypanosoma cruzi Genes That Encode Proteins of the Glycosylphosphatidylinositol Biosynthetic Pathway
Source: PLoS Negl Trop Dis. 2013 Aug 8;7(8):e2369. doi: 10.1371/journal.pntd.0002369 (PMC3738449; doi:10.1371/journal.pntd.0002369)

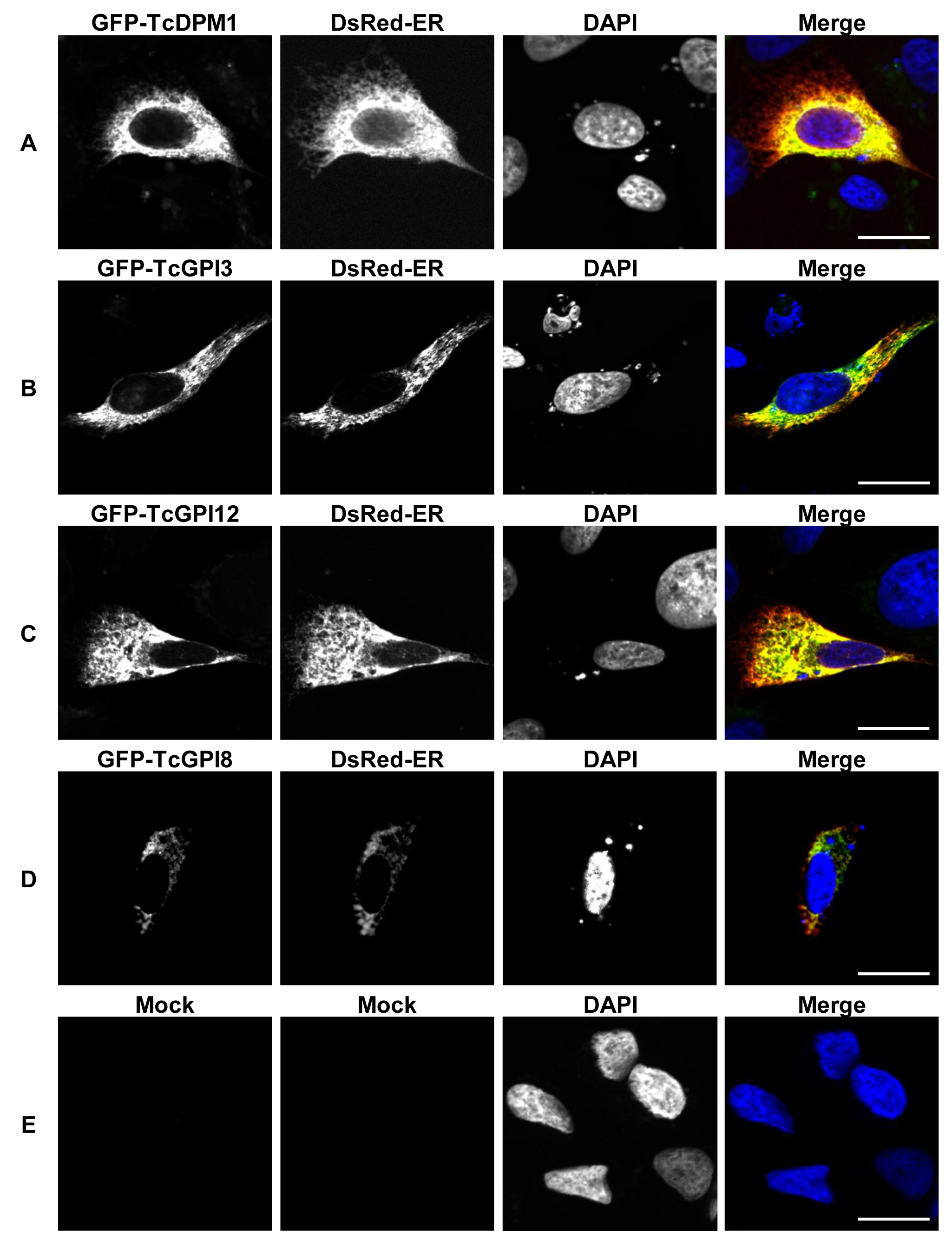

Supplement: Figure S1 — Cellular localization of T. cruzi proteins expressed in mammalian cells. The T. cruzi genes TcDPM1, TcGPI3, TcGPI12, and TcGPI8 were cloned in fusion with GFP in the vector pcDNA3.1/NT-GFP-TOPO and transfected into HT1080 human fibrosarcoma cells. Forty eight hours after transfections with pcDNA-GFP-TcDPM1 (A), pcDNA-GFP-TcGPI3 (B), pcDNA-GFP-TcGPI12 (C), pcDNA-GFP-TcGPI8 (D) or after mock transfections (E), cells were stained with DAPI and visualized under fluorescence microscopy. All plasmids were cotransfected with the pGAG-DsRed-ER plasmid to visualize cellular ER compartments. Scale bars: 20 µm. (TIF) [file pntd.0002369.s001.tif]

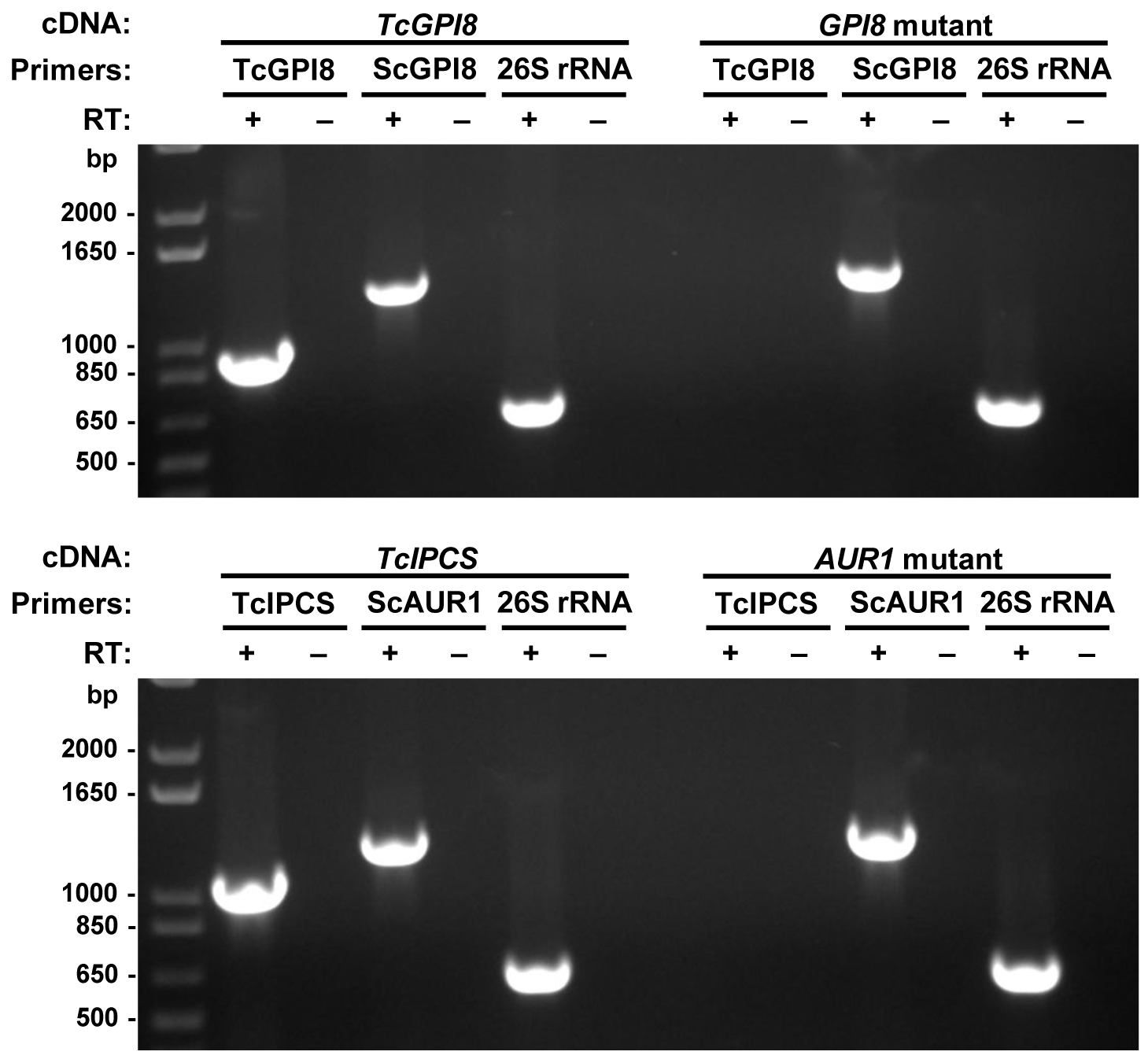

Supplement: Figure S2 — RT-PCR mRNA analysis of yeast mutants transformed with T. cruzi genes. Reverse-transcription and PCR amplifications (RT-PCR) of total RNA isolated from non-transformed yeast mutants or mutants transformed with T. cruzi genes were analyzed by agarose gel electrophoresis. Total RNA was isolated from GPI8 yeast mutants (top panel) or AUR1 mutants (bottom panel). mRNA expression was analyzed in non-transformed mutants (GPI8 mutants or AUR1 mutants) or mutants transformed with pRS426Met plasmids carrying either the T. cruzi (TcGPI8 or TcIPCS) that were grown in galactose-containing media. For each RNA sample, pair of primers used for cDNA amplifications, which are specific for the TcGPI8, TcIPCS, the endogenous ScGPI8 or ScAUR1, as well as for the yeast 26S rRNA genes, are indicated above each lane of the gel and are listed in Table S1. It is also indicated above each lane, whether the amplicons were generated in presence (+) or in the absence (−) of reverse transcriptase (RT). Molecular weight DNA markers are shown on the left. (TIF) [file pntd.0002369.s002.tif]

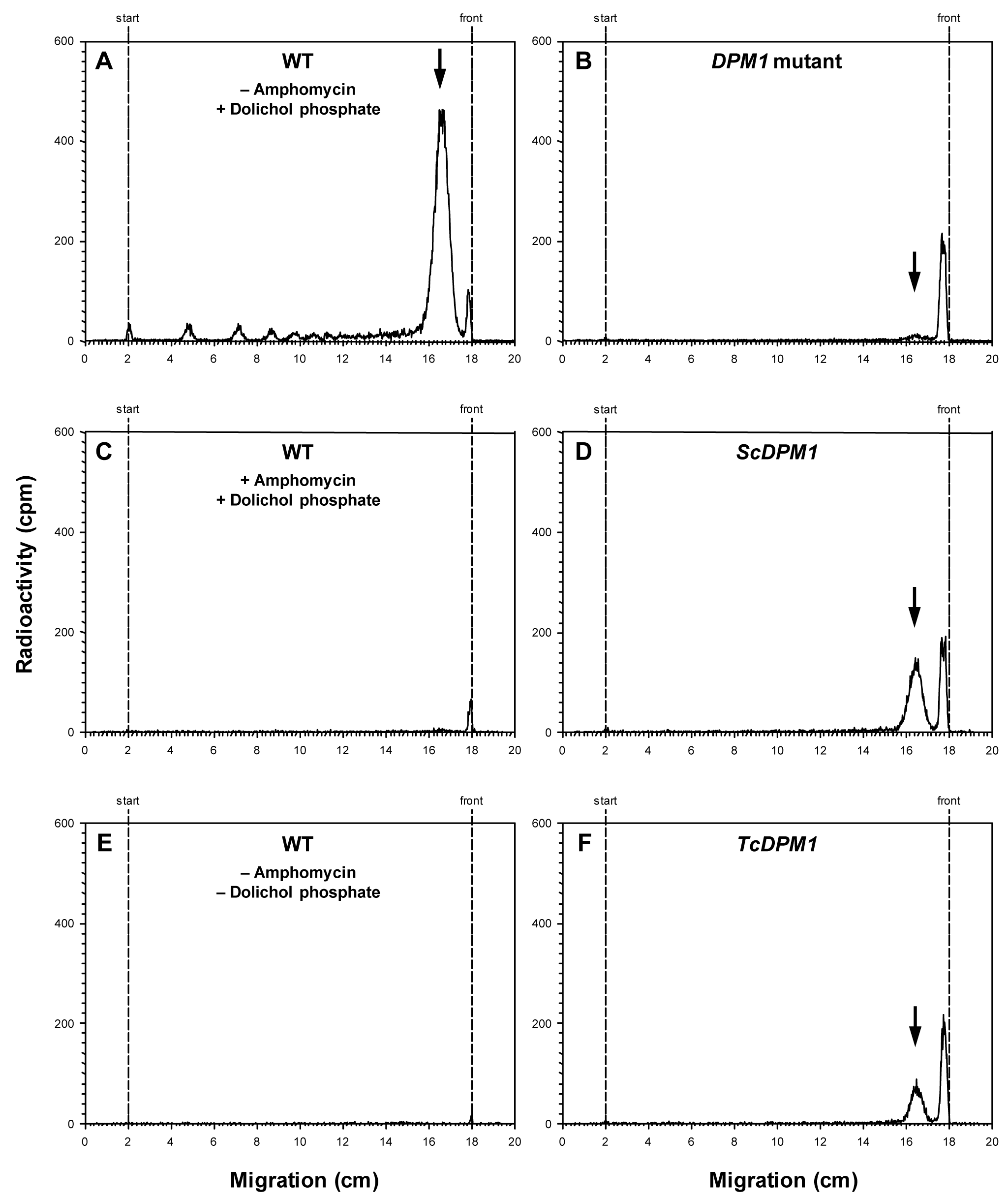

Supplement: Figure S3 — Synthesis of dolichol-P-mannose in yeast mutants expressing the TcDMP1 gene. Thin Layer Chromatography (TLC) of dolichol-phosphate-mannose in vitro labeled with GDP-[2-3H]mannose was performed using membrane fractions from: wild type yeast expressing the DPM1 endogenous gene (A), grown in the complete medium and preincubated with dolichol-phosphate; (B) DPM1 mutant grown in SD medium supplemented with uracil (nonpermissive conditions); (C) wild type yeast, expressing the DPM1 endogenous gene, grown in the YPGR medium and preincubated with amphomycin and dolichol-phosphate; (D) DPM1 mutant transformed with the recombinant plasmid pRS426Met containing the ScDPM1 grown in nonpermissive medium; (E) WT yeast, containing the DPM1 endogenous gene, grown in complete but not preincubated with amphomycin and dolichol-phosphate; (F) DPM1 mutant transformed with the recombinant plasmid pRS426Met containing the TcDPM1 grown in nonpermissive medium. The position of the dolichol-P-mannose (Dol-P-Man) in the TLC is indicated by an arrow. (TIF) [file pntd.0002369.s003.tif]

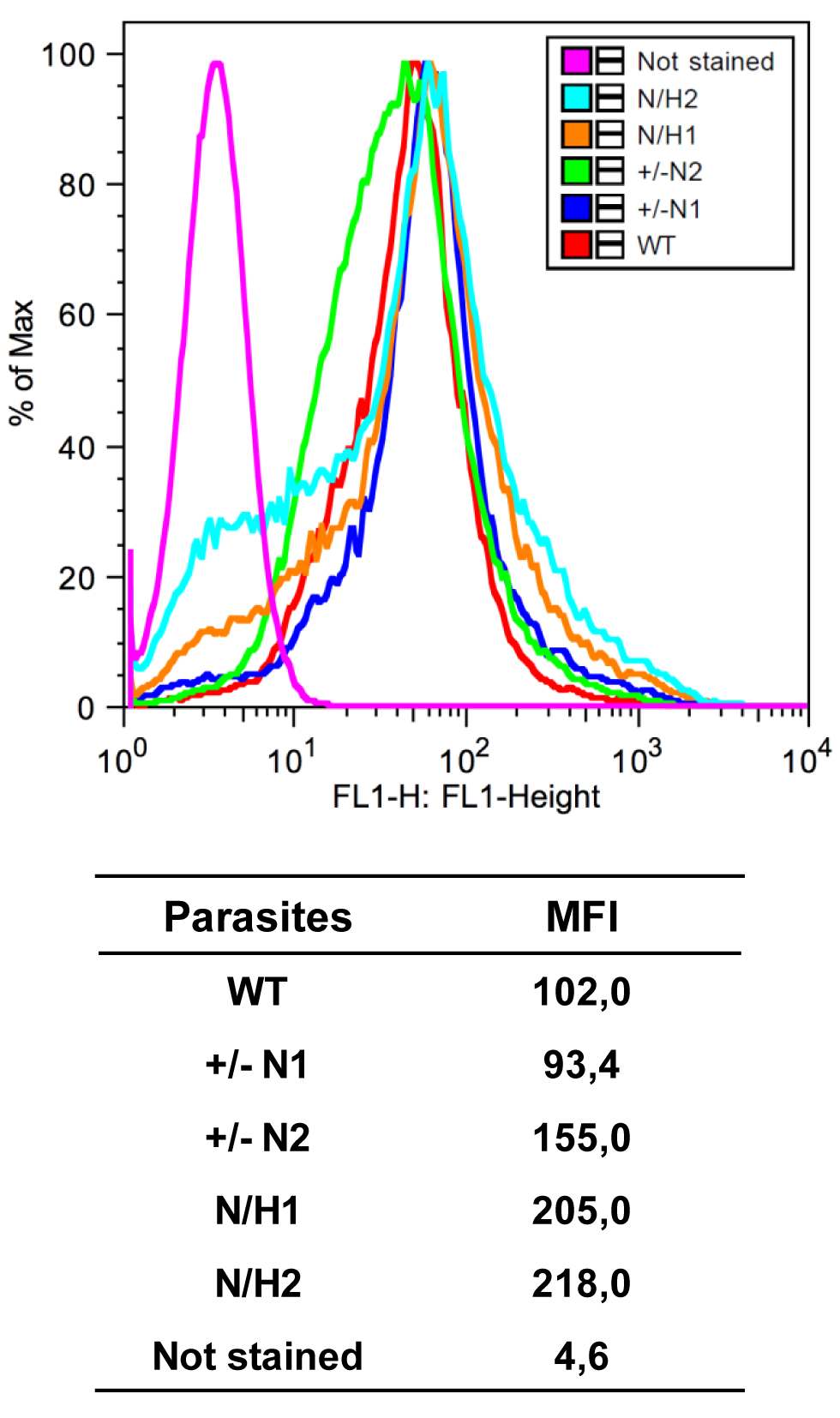

Supplement: Figure S4 — Flow cytometry analyses of T. cruzi mutants. Wild type epimastigotes (WT), two TcGPI8 single knockouts NeoR (+/− N1 and +/− N2) and double resistant clones (N/H1 and N/H2) were stained with the anti-mucin monoclonal antibody 2B10 (dilution 1∶450) and analyzed by flow cytometry. The values of mean fluorescence intensity (MFI) for each parasite cell line are shown below. (TIF) [file pntd.0002369.s004.tif]
