# Supplementary material for: Identification and Functional Analysis of Trypanosoma cruzi Genes That Encode Proteins of the Glycosylphosphatidylinositol Biosynthetic Pathway
Source: PLoS Negl Trop Dis. 2013 Aug 8;7(8):e2369. doi: 10.1371/journal.pntd.0002369 (PMC3738449; doi:10.1371/journal.pntd.0002369)
Supplement: Table S1 — Sequences of oligonucleotides used for PCR amplications and to generate plasmid constructs. (PDF) [file pntd.0002369.s005.pdf]

Primers	Sequences
**RT-PCR (yeast mutants)**	**TcGPI8 F:** 5' CTCGAATTCATGAGCAACAAAACAAAGACAAAC 3'
	**TcGPI8 R:** 5' GCTCTCGAGCAGCAGGTCATATTGTACATC 3'
	**TcIPCS F:** 5' GGGGAATTCATGGTTTTAATGGGGCCTCATTC 3'
	**TcIPCS R:** 5' TCTCTCGAGTCACCAGTGTCCAAAATTCCCAC 3'
	**ScGPI8 F:** 5' TTTGGATCCATGCGTATAGCGATGCATCT 3'
	**ScGPI8 R:** 5' GGCAAGCTTTTAATTAGTGTACAGGTCATA 3'
	**ScAUR1 F:** 5' TCTACTAGTATGGCAAACCCTTTTTCGAGATG 3'
	**ScAUR1 R:** 5' GCGAAGCTTTTAAGCCCTCTTTACACCTAGTG 3'
	**26 rRNA F:** 5' GCATATCAATAAGCGGAGGAAAAG 3'
	**26 rRNA R:** 5' GGTCCGTGTTTCAAGACGG 3'
**RT-PCR (parasites)**	**GPI8int 5'R2:** 5' ATGAGAGTTTTCATCCGTGTTGAG 3' (cDNA)
	**GPI8int 5'R:** 5' GGCGTATATTAAAGAAGTAGCGTGA 3' (PCR)
	**TcSL:** 5' ACAGTTTCTGTACTATATTG 3' (PCR)
**Transformation of yeast mutants**	**pRS TcDPM1 F:** 5' CGGATCCATGCCAATCAGCTACAC 3'
	**pRS TcDPM1 R:** 5' AAGCTTCTACACGACCTGTTTCAC 3'
	**pRS TcGPI3 F:** 5' GGGATCCATGGGAAGACACCGCGTG 3'
	**pRS TcGPI3 R:** 5' AAAGCTTTCAAACACCCTTCTCAAGAG 3'
	**pRS TcGPI8 F:** 5' CCCGGGATGAAGCGTCAGATGGG 3'
	**pRS TcGPI8 R:** 5' CAAGCTTCTACAGCAGGTCATATTG 3'
	**pRS TcGPI10 F:** 5' GGGGAATTCATGTCTCCTTGTATTGTGTATTGTTTCCCT 3'
	**pRS TcGPI10 R:** 5' GGGCTCGAGTTAATTGGCCTGAACAACATTTCGTA 3'
	**pRS TcGPI12 F:** 5' GGGATCCATGTCCGTTTTTTTCGCTG 3'
	**pRS TcGPI12 F:** 5' TAAAGCTTCTAGGACGCCTTCAGTTC 3'
	**pRS TcGPI14 F:** 5' GGGGAATTCATGCCGTGCCAGTCAGATTGTTCGTGGATC 3'
	**pRS TcGPI14 R:** 5' GGGCTCGAGCTACGCGAATTTGGTCATCTTTTTCCC 3'
	**pRS TcGAA1 F:** 5' GGATCCATGTCGATTGCCTTTTGC 3'
	**pRS TcGAA1 R:** 5' AAGCTTACATTGTTGGTAAAACTGATC 3'
	**pRS TcIPCS F:** 5' GGGGAATTCATGGTTTTAATGGGGCCTCATTC 3'
	**pRS TcIPCS R:** 5' TCTCTCGAGTCACCAGTGTCCAAAATTCCCAC 3'
	**pRS ScDPM1 F:** 5' GGGACTAGTATGAGCATCGAATACTCTGT 3'
	**pRS ScDPM1 R:** 5' GGGGGATCCTTAAAAGACCAAATGGTATAG 3'
	**pRS ScGPI3 F:** 5' GGGACTAGTATGGGCTTCAATATAGCGTATGTC 3'
	**pRS ScGPI3 R:** 5' CCCCTCGAGAGTTTCTCTTGCTTCCTTCGTCTC 3'
	**pRS ScGPI8 F:** 5' TTTGGATCCATGCGTATAGCGATGCATCT 3'
	**pRS ScGPI8 R:** 5' GGCAAGCTTTTAATTAGTGTACAGGTCATA 3'
	**pRS ScGPI10 F:** 5' GGGACTAGTATGGCTCACGAGGTTCATAGAATAAA 3'
	**pRS ScGPI10 R:** 5' GGGCTCGAGAATATCTGCCGCTGGAATATCGCTAT 3'
	**pRS ScGPI12 F:** 5' GGGACTAGTATGAAGATGTTGAGGCGTACAAAGGT 3'
	**pRS ScGPI12 R:** 5' GGGCTCGAGATATGTATAAACATCAAATTCATTAA 3'
	**pRS ScGPI14 F:** 5' GGGACTAGTATGACTGGCGAAGAATGGGGCTTGA 3'
	**pRS ScGPI14 R:** 5' GGGCTCGAGGTTGTTCTTTTTGTTGGAAACTGTGG 3'
	**pRS ScGAA1 F:** 5' TTTGGATCCATGGCCTTATTGGAGAAGTT 3'
	**pRS ScGAA1 R:** 5' GGCAAGCTTCTAACTTTGCTTTTCTTTCGA 3'
	**pRS ScAUR1 F:** 5' TCTACTAGTATGGCAAACCCTTTTTCGAGATG 3'
	**pRS ScAUR1 R:** 5' GCGAAGCTTTTAAGCCCTCTTTACACCTAGTG 3'
**Cellular localization in *T. cruzi***	**pTREX TcDPM1 F:** 5' GGGTCTAGAATGCCAATCAGCTACACCATAG 3'
	**pTREX TcDPM1 R:** 5' CCCGAATTCCACGACCTGTTTCACAGCCC 3'
	**pTREX TcGPI3 F:** 5' GGGTCTAGAATGGGAAGACACCGCGTGGCACT 3'
	**pTREX TcGPI3 R:** 5' CCCGAATTCAACACCCTTCTCAAGAGCATTAA 3'
	**pTREX TcGPI12 F:** 5' GGGTCTAGAATGTCCGTTTTTTTCGCTGTGAC 3'
	**pTREX TcGPI12 R:** 5' CCCGAATTCGGACGCCTTCAGTTCCTCTAGCT 3'
**Cellular localization in mammalian cells**	**pcDNA TcDPM1 F:** 5' GGGGCTAGCTGCCAATCAGCTACACCATAG 3'
	**pcDNA TcDPM1 R:** 5' CCCTCTAGACACGACCTGTTTCACAGCCC 3'
	**pcDNA TcGPI3 F:** 5' GGGGGTACCGGGAAGACACCGCGTGGCACTGG 3'
	**pcDNA TcGPI3 R:** 5' CCCTCTAGAAACACCCTTCTCAAGAGCATTAA 3'
	**pcDNA TcGPI12 F:** 5' GGGGCTAGCTGTCCGTTTTTTTCGCTGTGAC 3'
	**pcDNA TcGPI12 R:** 5' CCCTCTAGAGGACGCCTTCAGTTCCTCTAGC 3'
	**pcDNA TcGPI8 F:** 5' GGGGCTAGCTGAAGCGTCAGATGGGGGTTTTG 3'
	**pcDNA TcGPI8 R:** 5' CCCTCTAGACAGCAGGTCATATTGTACATCCA 3'
**Knockout constructions**	**GPI8/*Sac* I 5'F:** 5' TGAGCTCTGGGGAGTGAGGGTGGCTGCGAG 3'
	**GPI8/*Spe* I 5'R:** 5' ACTAGTAACTCTCCTATGATGTCGTCTTTG 3'
	**GPI8/*Xho* I 3'F:** 5' TCTCGAGCATGCAAAGTGTGAAGTCGGACC 3'
	**GPI8/*Xba* I 3'R:** 5' CTCTAGATTCCTTGTTGCTTTGCTCTTATC 3'
	**GPI8-Al2/KpnI 5'F:** 5' TTTGGTACCCTTGTCACATGCCATTTTCCA 3'
	**GPI8-Al2/SpeI 5'R:** 5' GGGACTAGTGCAGAGATGGTAAATTGTCAGT 3'
	**GPI8-Al2/EcoRV 3'F:** 5' GGGGATATCCTCACGCTGGTCACTTGAATC 3'
	**GPI8-Al2/XhoI 3'R:** 5' TTTCTCGAGTGAGCTGCATGCTTCCAAGC 3'
	**GPI8 ext 5'F:** 5' GTAACACAATCGTTTTTAGGCTGTG 3' (Primer 1F)
	**GPI8 ext 3'R:** 5' CAGATGAGGAAAGAGACGAACAG 3' (Primer 2R)
	**GPI8 int 5'R:** 5' GGCGTATATTAAAGAAGTAGCGTGA 3' (Primer 7R)
	**GPI8 int 5'R2:** 5' ATGAGAGTTTTCATCCGTGTTGAG 3' (Primer 8R)
	**Neo For:** 5' CGACCCTGCAGCCAATATGGGATC 3' (Primer 3F)
	**Neo Rev:** 5' TCAGAAGAACTCGTCAAGAAGGCG 3' (Primer 3R)
	**Neon11:** 5' CGGCCGGAGAACCTGCGTGCAA 3' (Primer 1R)
	**Neon20:** 5' GGATTCATCGACTGTGGCCGGC 3' (Primer 2F)
	**Hyg For:** 5' ATGAAAAAGCCTGAACTCACCGC 3' (Primer 6F)
	**Hyg Rev:** 5' TCAGAAGAACTCGTCCGGCCACA 3' (Primer 6R)
	**Hygn11:** 5' GAGCTGCATCAGGTCGGAGACGC 3' (Primer 4R)
	**Hygn20:** 5' ACCGATGGCTGTGTAGAAGTAC 3' (Primer 5F)
